# Cortical preparatory activity indexes learned motor memories

**DOI:** 10.1038/s41586-021-04329-x

**Published:** 2022-01-26

**Authors:** Xulu Sun, Daniel J. O’Shea, Matthew D. Golub, Eric M. Trautmann, Saurabh Vyas, Stephen I. Ryu, Krishna V. Shenoy

**Affiliations:** 1 Department of Biology, Stanford University, Stanford, CA, USA.; 2 Wu Tsai Neurosciences Institute, Stanford University, Stanford, CA, USA.; 3 Department of Electrical Engineering, Stanford University, Stanford, CA, USA.; 4 Department of Bioengineering, Stanford University, Stanford, CA, USA.; 5 Department of Neurosurgery, Palo Alto Medical Foundation, Palo Alto, CA, USA.; 6 Department of Neurosurgery, Stanford University, Stanford, CA, USA.; 7 Department of Neurobiology, Stanford University, Stanford, CA, USA.; 8 Howard Hughes Medical Institute, Stanford University, Stanford, CA, USA.; 9 These authors contributed equally: Xulu Sun, Daniel J. O’Shea.

## Abstract

The brain’s remarkable ability to learn and execute various motor behaviours harnesses the capacity of neural populations to generate a variety of activity patterns. Here we explore systematic changes in preparatory activity in motor cortex that accompany motor learning. We trained rhesus monkeys to learn an arm-reaching task^1^ in a curl force field that elicited new muscle forces for some, but not all, movement directions^2,3^. We found that in a neural subspace predictive of hand forces, changes in preparatory activity tracked the learned behavioural modifications and reassociated^4^ existing activity patterns with updated movements. Along a neural population dimension orthogonal to the force-predictive subspace, we discovered that preparatory activity shifted uniformly for all movement directions, including those unaltered by learning. During a washout period when the curl field was removed, preparatory activity gradually reverted in the force-predictive subspace, but the uniform shift persisted. These persistent preparatory activity patterns may retain a motor memory of the learned field^5,6^ and support accelerated relearning of the same curl field. When a set of distinct curl fields was learned in sequence, we observed a corresponding set of field-specific uniform shifts which separated the associated motor memories in the neural state space^7–9^. The precise geometry of these uniform shifts in preparatory activity could serve to index motor memories, facilitating the acquisition, retention and retrieval of a broad motor repertoire.

Motor learning encompasses a wide range of phenomena, from low-level calibration of movement parameters to high-level cognitive decisions in action selection^10^. Motor adaptation is a form of motor learning by which motor commands are modified to achieve desired movements in a new environment. Decades of studies have explored the behavioural principles of motor adaptation, describing the process of error-driven movement calibration, the generalization of learned skills across contexts, memory retention and savings, and interference between multiple skills^2,3,7,8,10–13^. However, the neural mechanisms that support these diverse motor learning phenomena remain poorly understood.

One emerging approach to understanding these neural computations is through the study of neural population dynamics, which has provided insight into complex activity patterns that defy understanding at the level of individual neurons^14–16^. Recently, this framework has begun to elucidate the neural foundation of motor learning at the population level^17–20^. Preparatory neural activity that precedes movement serves to initialize the pattern-generating neural population dynamics that control movement. To support motor learning, preparatory neural states and the subsequent neural population dynamics must adapt to modify outgoing motor commands. Consequently, we expect that some changes in preparatory activity that accompany motor learning would be tethered to changes in motor output^18,20^. Moreover, we propose that additional changes in neural preparatory activity, not directly coupled to movement output, might also emerge during learning. Such changes might facilitate learning and retention by organizing the population dynamics that implement new motor behaviours. We sought to test these hypotheses using a curl force field motor learning task.

## Motor adaptation in a curl field task

**Extended Data Fig. 1 |:**
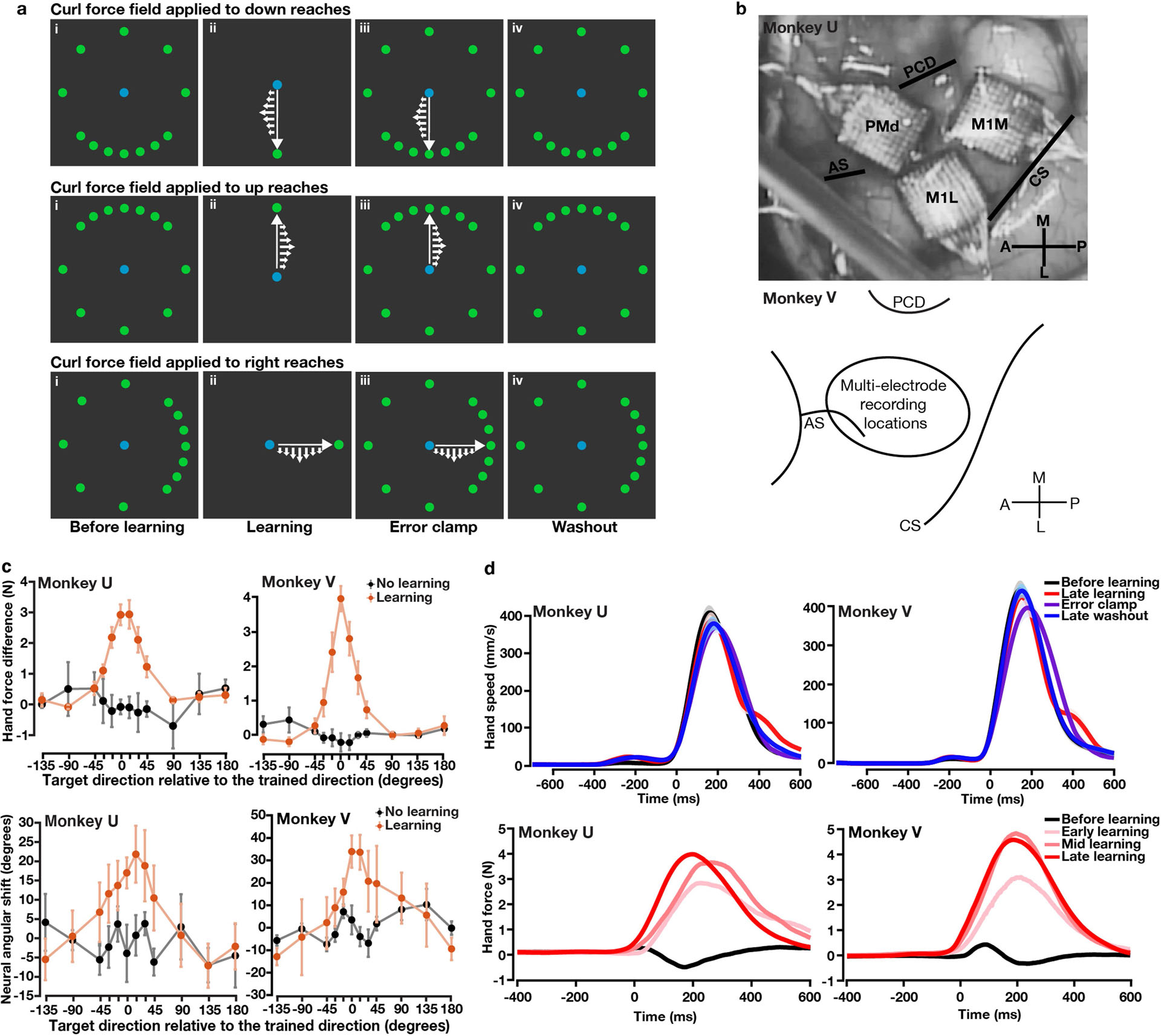
Additional information for task design, recording sites and behavioural performance. **a**, Spatial arrangements of the 12 reaching targets. The target density near the trained target (down, up or right) was higher in order to sample more neural states for reaches that were more likely to be altered by learning, for the purpose of studying generalization of learning^3^. Ideally, one would like to have equally-spaced reach targets as dense as possible, but because monkeys could perform a limited number of trials each day, a compromise solution was to increase the density of targets near the trained target. Note that the curl field can be either clockwise (CW) or counterclockwise (CCW). Here we show CW fields as an example. **b**, Utah-array implant locations in monkey U and recording sites in monkey V. Recordings were performed in PMd and M1 in the hemisphere contralateral to the reaching arm. Top panel: an intraoperative photo of three Utah-array implants in monkey U. Bottom panel: a schematic map illustrating the approximate locations of recording sites in monkey V based on stereotactic coordinates; data in this work included units recorded from multi-electrode V-probes and Neuropixels probes. Histology has not yet been done on either monkey. Using the cortical landmarks, we estimated that the recording sites in monkey V largely overlapped with the lateral half of the area covered by the three Utah arrays in monkey U. AS: Spur of arcuate. CS: central sulcus. PCD: precentral dimple. **c**, We computed behavioural (top panel) and neural generalization (bottom panel) with the sign of the effects flipped for CCW fields to match the effects of CW fields, compared to Fig. 1d and Fig. 2c. We found a spatial asymmetry in behavioural and neural generalization, with more learning in the ‘push’ direction (i.e., the direction to oppose the curl field). Error bars, s.e.m. across sessions (monkey U, n = 4, 3; monkey V, n = 5, 3). **d**, Top panel: trial-averaged hand speed in different blocks over multiple learning sessions. Shaded area, s.e.m. across sessions (monkey U, n = 4; monkey V, n = 5). Bottom panel: compensatory hand force perpendicular to the reach direction in one example session. Hand force in late-learning trials (dark red) showed a more stereotypical, less variable temporal pattern with an earlier onset than in early-learning trials (light red). Time zero, movement onset.

**Fig. 1 |:**
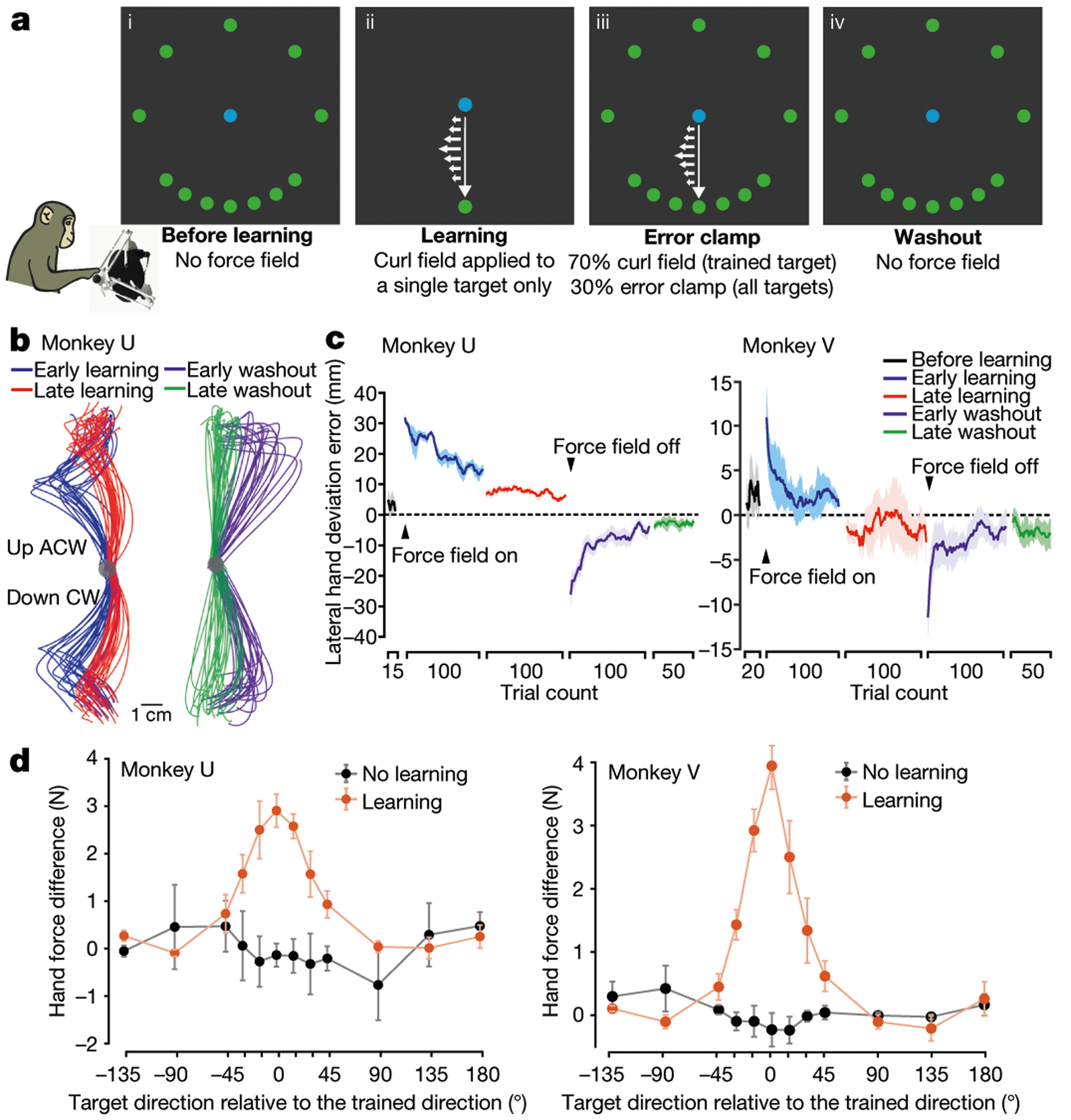
Task design and behavioural performance. **a**, Block schematic of the curl force field learning task (blue, workspace centre; green, reach targets). A curl field (small white arrows) was applied at a single trained target; generalization of learning to other targets was probed via error clamp. Bottom left, an illustration of a monkey controlling a haptic device. **b**, Hand trajectories during learning and washout from two representative sessions. CW, clockwise; ACW, anticlockwise. **c**, Behavioural learning and washout measured as lateral hand deviation. **d**, Perpendicular hand force difference between error-clamp trials and before-learning trials showed local generalization (orange), in contrast to no-learning control sessions (black). Data are mean ± s.e.m. across sessions.

We trained two rhesus monkeys (U and V) to perform an instructed-delay reaching task that elicited adaptation to counteract a curl force field (Fig. 1a, Extended Data Fig. 1a). Before learning, monkeys made straight centre-out reaches towards each of 12 targets by controlling a haptic device. In the learning block, monkeys reached towards a single trained target while the device applied a curl force field that was perpendicular to movement direction and proportional to hand speed. Late in learning, the curl field remained active for reaches to the trained target, which were interleaved with reaches to all 12 targets with an error clamp rendered by the device. The error clamp constrained movements to a straight line towards the target, hence clamping error feedback to zero to assess the feed-forward learning of the curl field. Finally, in the washout block, the curl field and error clamp were removed to probe the after-effects of learning and de-adaptation.

Monkeys displayed gradual behavioural learning and washout, performing straighter reaches with reduced lateral deviation (Fig. 1b, c). The error clamp revealed a bell-shaped spatial pattern of generalization where the strength of learning-induced force changes fell off with increasing angular distance from the trained target (Fig. 1d), consistent with human behavioural studies^2,3,11^.

## A neural subspace tracks generalization

**Extended Data Fig. 2 |:**
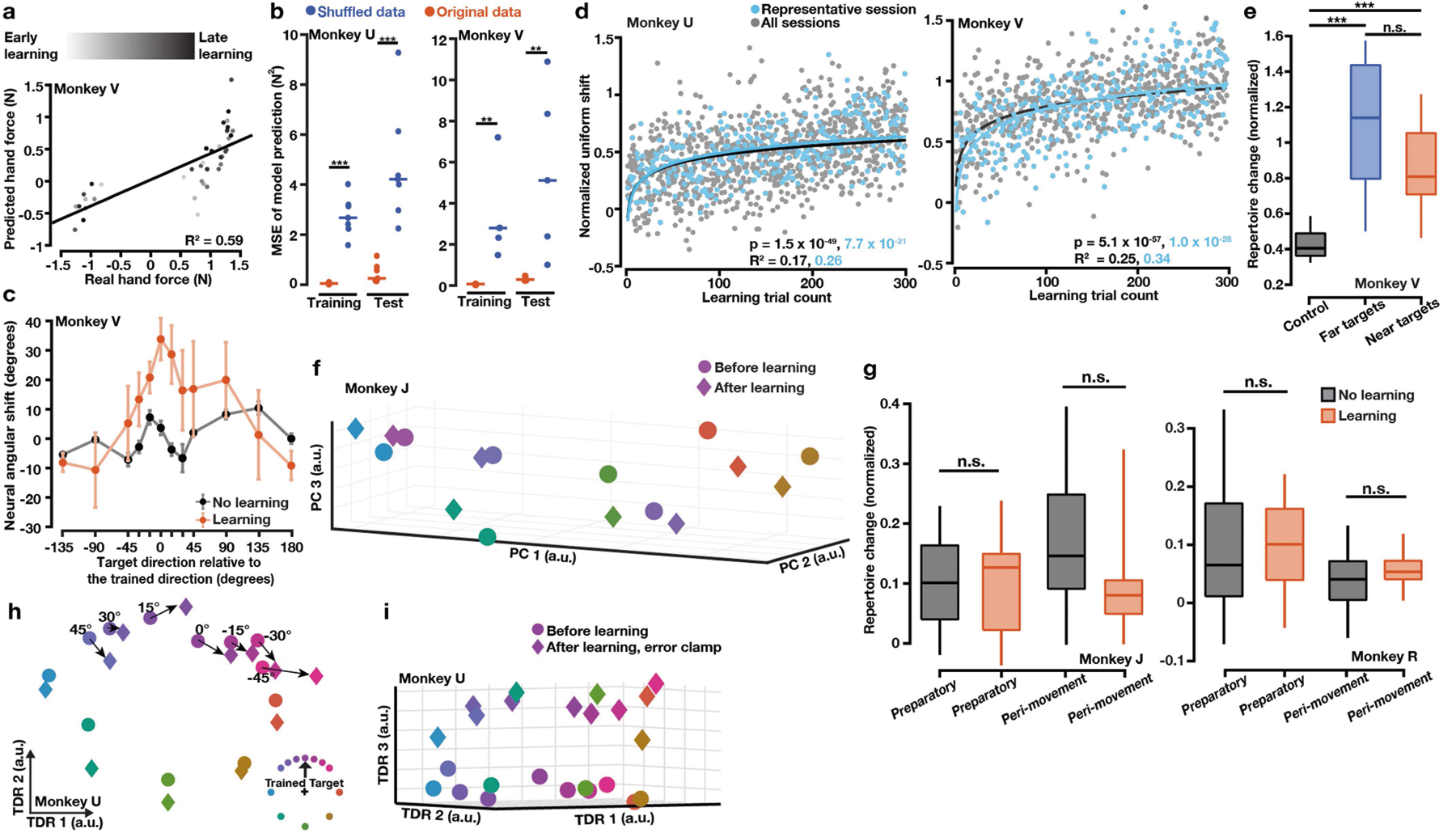
Additional results of neural activity patterns during curl field learning and VMR learning. **a**, Initial hand forces predicted by the 2D TDR preparatory states were correlated with real forces of the upcoming movement (the slope is 0.41 and the intercept is 0.02 with R^2^ = 0.59 and *P* = 6.06 × 10^−9^). The sign of hand force indicates its direction. Lighter dots, earlier learning trials; darker dots, later learning trials. **b**, Single-trial prediction MSE of initial hand forces was significantly smaller using original data than shuffled data (two-sided Wilcoxon rank-sum test: *P* = 0.0006 for both training and test sets of monkey U and *P* = 0.008 for both training and test sets of monkey V). Training set: before-learning trials. Test set: learning trials. Control results (blue) were forces predicted by models built from training sets that had neural and behavioural data shuffled. One datapoint per session. **c**, Changes of preparatory states in the force-predictive TDR subspace reflected generalization of learning, quantified as the rotatory angle from before-learning to error-clamp neural states. Zero degree on the x axis, the trained target. Error bars, s.e.m. across sessions (n = 5, 3). **d**, Normalized single-trial neural shift during learning along the uniform-shift learning axis. Solid line: linear-log regression (n = 1200, 900). **e**, Preparatory neural repertoires changed similarly for trained and untrained reaches. Black: no-learning control results (n = 36). Blue: far targets more than 45 degrees from the trained target (n = 15). Red: near targets within 45 degrees from the trained target (n = 21). One-sided Wilcoxon rank-sum test: *P*_*black vs. blue*_ = 2.33 × 10^−7^, *P*_*black vs. red*_ = 4.74 × 10^−8^, *P*_*blue vs. red*_ = 0.059. **f, g**, VMR learning results. **f**, Preparatory neural states projected to PCs 1–3. After-learning states (diamonds) were mixed with before-learning states (circles). One example session. **g**, Preparatory and peri-movement neural activity patterns did not show repertoire change during VMR learning. One-sided Wilcoxon rank-sum test: *P* > 0.1 for all comparisons. Three learning sessions (n = 24) and three control sessions (n = 24) for both monkeys. **h, i**, Neural preparatory states in the 3D TDR subspace. The 3D subspace was constructed by TDR capturing the variance due to initial hand forces and a binary indicator of trial conditions (an indicator of before-learning versus after-learning). One example session. **h**, In the force-predictive TDR subspace, rotatory shifts of preparatory neural states were similar to Fig. 2b. **i**, Along the TDR 3 axis (the binary indicator axis), this 3D model revealed a uniform shift similar to what we observed along PC 3 in the PCA subspace (Fig. 2d). For all the box plots, the central line indicates the median, the bottom and top edges indicate the 25th and 75th percentiles of the data, and the whiskers extend to the 5th and 95th percentiles of the data.

**Fig. 2 |:**
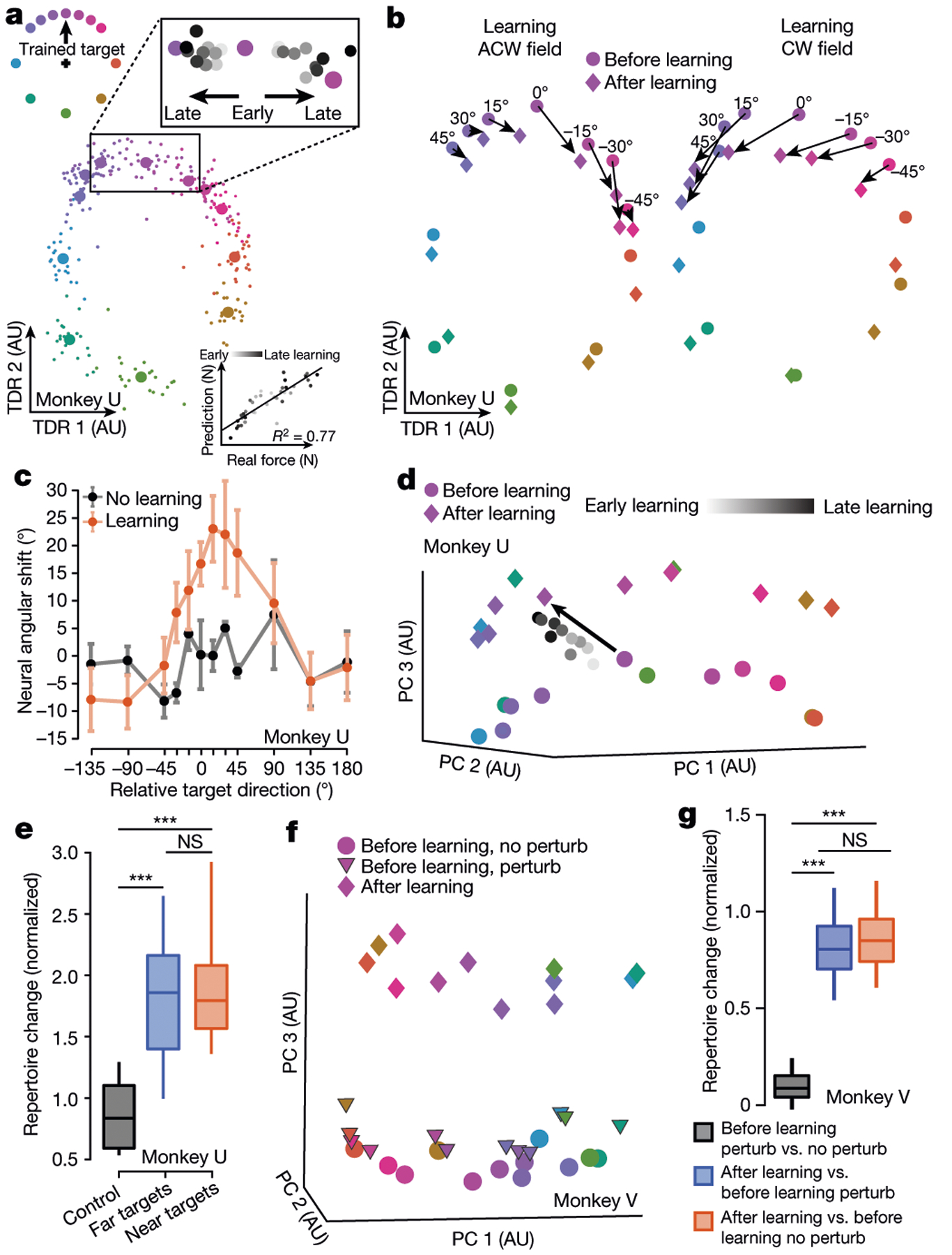
Changes in preparatory neural states accompanying learning. **a**, In the force-predictive TDR subspace, single trial (small circles) and condition-averaged (large circles) before-learning preparatory states were radially organized. Inset shows progression of preparatory states during learning (grey to black) in two example learning sessions with anticlockwise and clockwise fields. Bottom right, preparatory states within this space predicted initial hand forces. AU, arbitrary units. **b**, **c**, Neural correlates of generalization in this TDR subspace probably reflected compensatory initial forces. Preparatory states for the trained target (0°) and nearby targets (within 45°) shifted after curl field learning but not in no-learning control sessions. Neural states visualized in **b** and angular shifts quantified in **c**. Error bars: s.e.m. across sessions. **d**, After-learning preparatory states for all targets shifted away from their before-learning states, visualized in the leading principal components (PCs). Grey circles, preparatory states for the trained target during learning. **e**, After-learning preparatory states comprised a new neural repertoire for all targets near and far from the trained target, in contrast to a lack of repertoire change in no-learning control sessions. **f**, **g**, Preparatory states shifted uniformly (**f**) and comprised a new neural repertoire (**g**) following learning but not during control reaches with random force perturbations (perturb). ***P < 0.001; NS, not significant. See Supplementary Table 1 for statistics.

We recorded neural activity in dorsal premotor (PMd) and primary motor (M1) cortices using Neuropixels probes, Utah arrays and V-probe linear arrays. Single-neuron activity during learning and washout was heterogeneous and complex, consistent with previous reports^5,19^. To search for structured changes in preparatory neural population activity accompanying learning^14,15,21^, we applied targeted dimensionality reduction^22^ (TDR) on before-learning trials, which identified a neural subspace in which preparatory states were predictive of initial hand forces in the upcoming movement. In this force-predictive subspace, before-learning neural states were radially organized by reach directions^18,23^ (Fig. 2a); during learning, preparatory states of the trained target rotated towards the preparatory state of the adjacent target opposite to the curl field direction (Fig. 2a, top-right inset). This rotatory progression probably reflected the preparation of initial compensatory forces to counter the curl field. Preparatory states in this subspace predicted the observed hand forces with high accuracy (Fig. 2a, bottom inset, Extended Data Fig. 2a, b).

Following learning, preparatory states for nearby, untrained targets also rotated towards the adjacent preparatory states (Fig. 2b). These rotatory neural state shifts followed a similar spatial profile as behavioural generalization (Fig. 2c, Extended Data Fig. 2c), which was bell-shaped around the trained target with spatial asymmetry (Extended Data Fig. 1c) and thereby constituted a neural correlate of motor learning generalization, as predicted by previous work^24^. These neural changes may reflect the state of an adapting internal model that maps between desired movements and neural commands. In this framework, generalization may result from a neural population code in which spatial basis functions are shared by reaches to nearby targets^25^. Adapting to a curl field at the trained target modifies this shared basis, thereby influencing untrained reaches in a spatially localized manner.

The learning-induced changes in preparatory states within this force-predictive subspace were closely coupled to changes in movement output. These changes are similar to the re-aiming strategy reported in visuomotor rotation (VMR) learning tasks, in which motor preparatory activity rotates in the opposite direction of the rotated visual feedback^18,26,27^. They are also consistent with a more general ‘reassociation’ strategy observed during short-term brain-computer interface (BCI) learning^4^. Within this neural subspace, the motor system may repurpose existing activity patterns, reflecting a common strategy across different motor learning contexts^4,18,26–30^.

## A uniform shift of neural population activity

**Extended Data Fig. 3 |:**
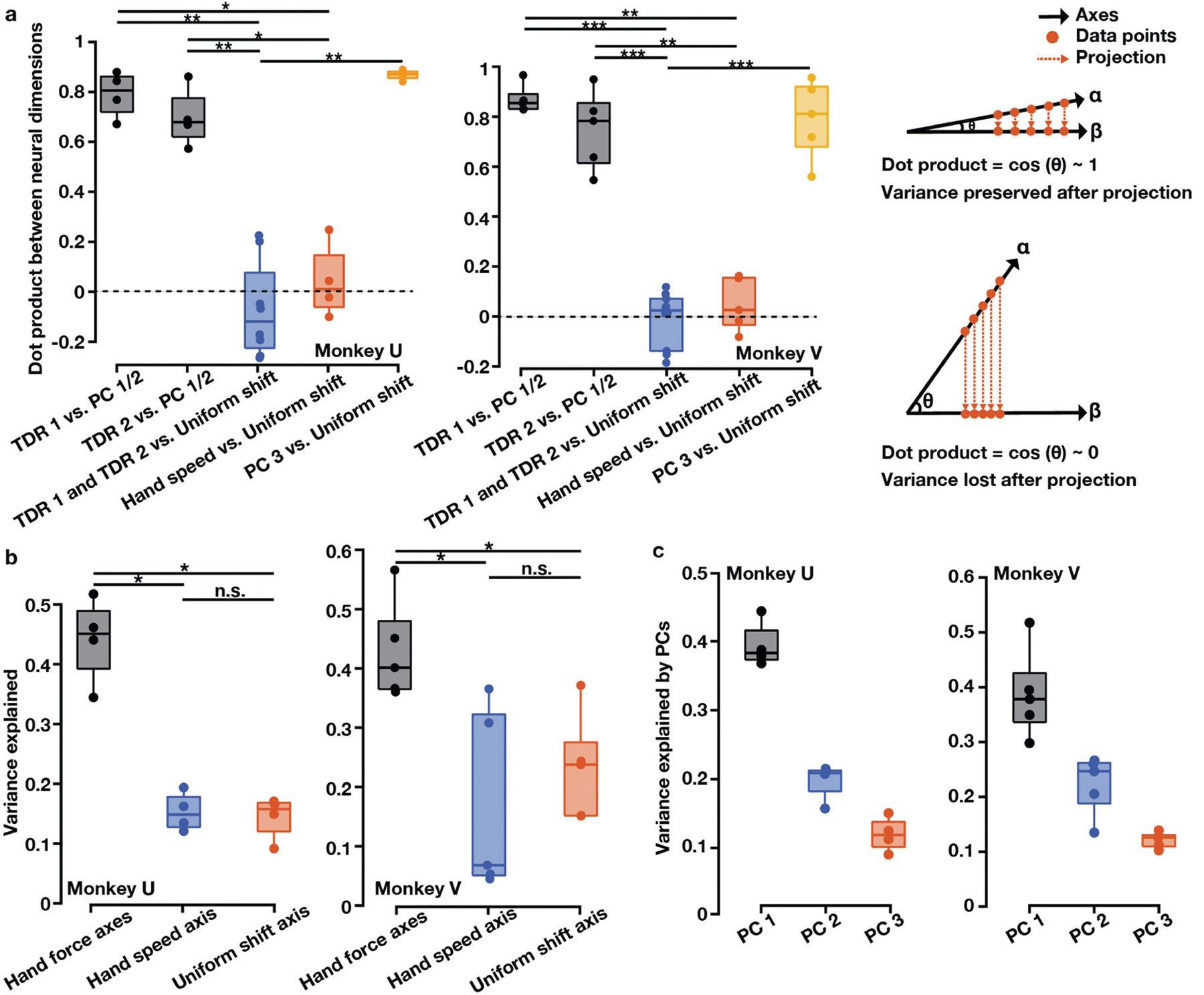
Relationships between neural population dimensions and total neural variance explained by different dimensions. **a**, Pairwise dot products between neural population dimensions. Values close to 1 indicate that two dimensions are closely aligned and values close to 0 indicate that two dimensions are nearly orthogonal. In each session, we calculated the dot product of TDR 1 and PC 1 and the dot product of TDR 1 and PC 2, and took the larger value of the two dot products (TDR 1 vs. PC 1 / 2). We then calculated the dot product of TDR 2 and the PC axis not used for multiplying with TDR 1 (TDR 2 vs. PC 1 / 2). The PC 1 / 2 plane largely overlapped with the TDR 1 / 2 plane (black). The TDR 1, TDR 2, and hand-speed TDR axes were all nearly orthogonal to the uniform-shift learning axis (blue and red). PC 3 largely overlapped with the uniform-shift learning axis (yellow). Two-sided Wilcoxon rank-sum test: monkey U, **P = 4.04 × 10^−3^, *P = 0.029; monkey V, ***P = 6.66 × 10^−4^, **P = 7.94 × 10^−3^. Right panel: a schematic illustration of projecting data points from axis *α* to axis *β* and the corresponding dot product. **b, c**, The portion of total neural activity variance explained by the TDR 1 and TDR 2 (hand force) axes, hand-speed TDR axis, uniform-shift axis and PCs 1–3. **b**, Two-sided Wilcoxon rank-sum test: monkey U, *P = 0.029, n.s. *P* = 1; monkey V, *P = 0.016 and 0.032, n.s. *P* = 0.42. **a–c**, n = 4 (monkey U) and n = 5 (monkey V). For all the box plots, the central line indicates the median, the bottom and top edges indicate the 25th and 75th percentiles of the data, and the whiskers extend to the 5th and 95th percentiles of the data.

**Extended Data Fig. 4 |:**
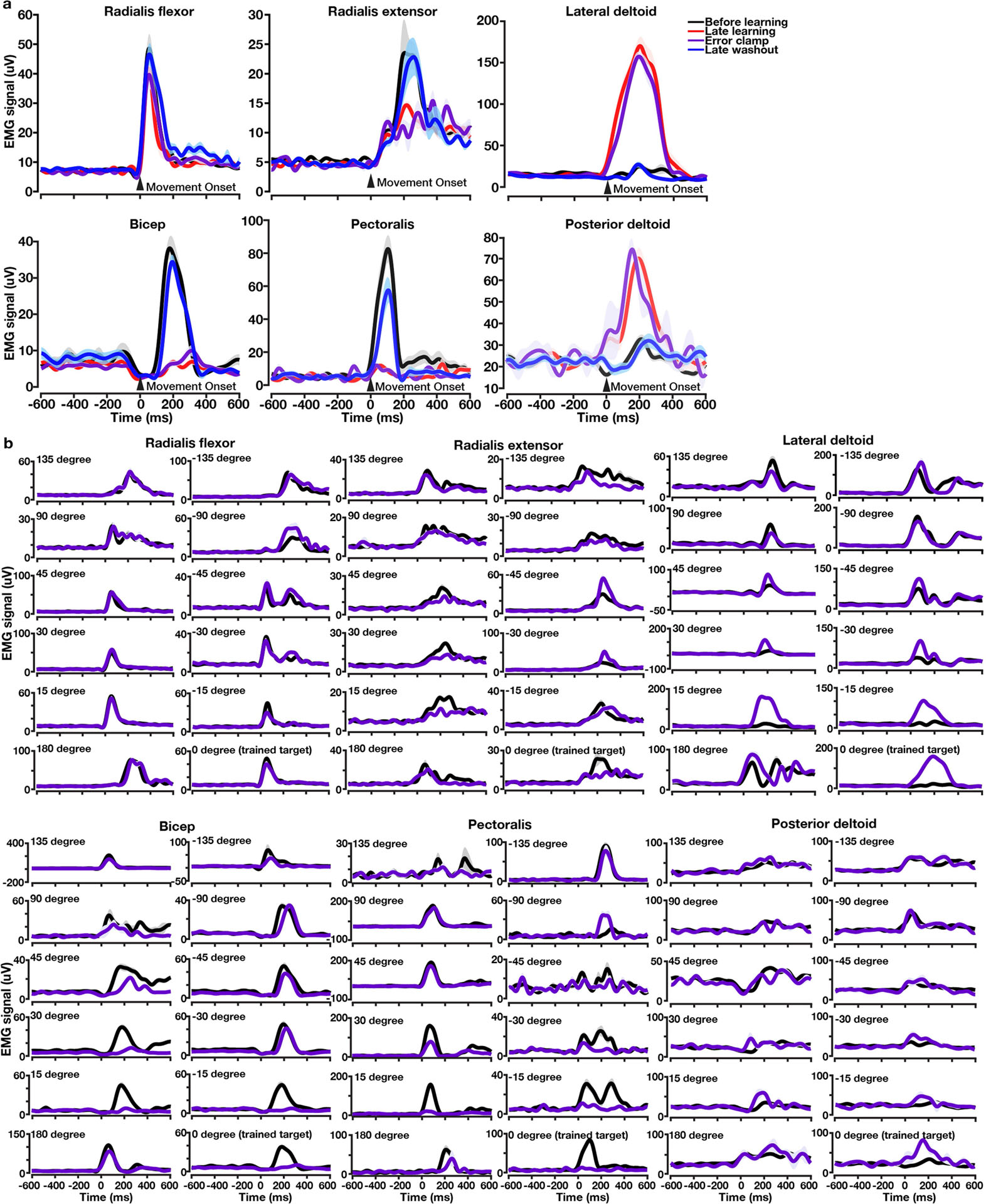
EMG signals of 6 upper limb muscles (bicep, radialis flexor, radialis extensor, pectoralis, posterior deltoid, lateral deltoid). Time zero, movement onset. One example condition (CW curl field applied to down reaches). Shaded area, s.e.m. across trials. **a**, EMG signals in before-learning, late-learning, error-clamp and late-washout blocks. Muscle activity did not show signs of muscle co-contraction during learning (red). Muscle activity during the preparatory period remained flat and around the same level across all blocks (two-sided rank-sum test: *P* < 0.0001 for comparing late-learning or error-clamp bicep activity with before-learning or late-washout bicep activity during the preparatory period; *P* > 0.3 for all the other pairs of comparison during the preparatory period). Muscle activity patterns in before-learning (black) and late-washout trials (blue) were very similar. Muscle activity patterns in late-learning (red) and error-clamp trials (purple) were very similar. **b**, EMG signals in before-learning (black) and error-clamp (purple) blocks did not show a uniform shift across all 12 reaching targets. For all six muscles, EMG activity after learning increased in some directions and decreased in other directions. Muscle activity of reaching to the target 135 degrees away from the trained target (i.e., far targets with almost no behavioural generalization, see Fig. 1c) in before-learning and error-clamp trials showed similar temporal patterns.

**Extended Data Fig. 5 |:**
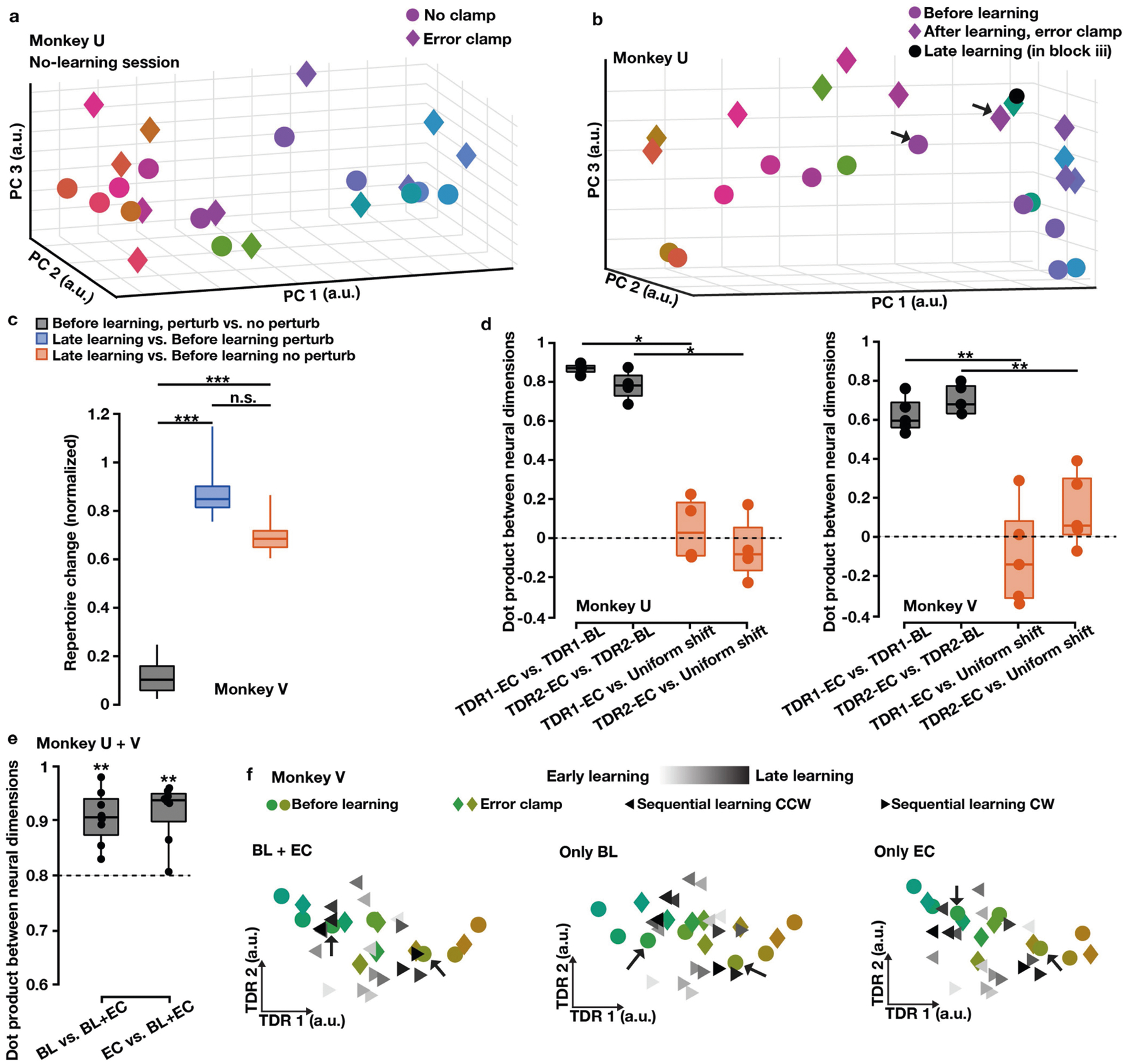
The uniform shift is not due to the error clamp. **a**, Preparatory states in error-clamp (diamonds) and no-clamp (circles) trials were not significantly different in no-learning control sessions (Hotelling’s T^2^ test: *P* > 0.5 for all control sessions of monkeys U and V). One example session is shown. **b**, In the error-clamp block (block iii) of learning sessions, the late-learning preparatory state and error-clamp state of the trained target were not significantly different (Hotelling’s T^2^ test: *P* > 0.05 for all learning sessions of monkey U and *P* > 0.1 for all learning sessions of monkey V). Arrows point to the before-learning state (purple circle) and error-clamp state (purple diamond) of the trained target. One example session is shown. **c**, Late-learning preparatory states comprised a new neural repertoire following learning but not during control reaches with random force perturbations. One-sided Wilcoxon rank-sum test: ***P = 1.83 × 10^−5^, n.s. *P* = 0.99 (n = 12 per box). The results are similar to Fig. 2g where we used after-learning, error-clamp trials to compute the repertoire change. **d–f**, The uniform shift was not due to reorientation of the TDR plane in error-clamp trials. **d**, TDR axes using only error-clamp trials (TDR-EC) and TDR axes using only before-learning trials (TDR-BL) were largely aligned. The error-clamp TDR 1 axis and before-learning TDR 1 axis were highly aligned, and same for TDR 2 axes (black). The error-clamp TDR axes were nearly orthogonal to the uniform-shift axis (red), similar to the before-learning TDR axes shown in Extended Data Fig. 3a. One-sided Wilcoxon rank-sum test: monkey U, n = 4 and *P = 0.014; monkey V, n = 5 and **P = 4.0 × 10^−3^. **e**, In the interference experiments, the force-predictive TDR planes constructed from only before-learning (BL) trials, only error-clamp (EC) trials, and both (BL+EC) were significantly aligned. One-sided signed-rank test comparing dot products to 0.8: n = 8 from monkeys U + V, **P = 3.9 × 10^−3^. **f**, In the interference experiments, preparatory neural states showed similar patterns in the force-predictive TDR plane built from only before-learning (BL) trials, only error-clamp (EC) trials, or both (BL+EC). One example session. Small black arrows point to the before-learning states of trained targets. For all the box plots, the central line indicates the median, the bottom and top edges indicate the 25th and 75th percentiles of the data, and the whiskers extend to the 5th and 95th percentiles of the data.

We next applied principal component analysis (PCA) to preparatory activity to probe for additional changes during learning. The first two principal components largely overlapped with the force-predictive subspace (Extended Data Fig. 3a); however, along the third principal component, preparatory states shifted uniformly during learning for all targets, including those far from the trained target (Fig. 2d). To determine a neural axis that captures this uniform shift in the full-dimensional neural space, we defined the uniform-shift axis as the vector connecting the centroid of before-learning states to the centroid of after-learning states (subtracting a consistently small component within the force-predictive TDR subspace; Extended Data Fig. 3a). Along this uniform-shift axis, preparatory states of the trained target shifted gradually during learning (Fig. 2d, Extended Data Fig. 2d). We performed a variety of control analyses that demonstrated that the uniform shift could not be attributed to learning-unrelated changes in behaviour, including speed, muscle activation, stiffness, posture and error-clamp movements (Extended Data Figs. 1d, 3–5), or to changes in neural tuning, including preferred direction and background drift^31^ (Supplementary Table 2, Supplementary Note 2). The uniform shift therefore may facilitate learning itself rather than mirror behavioural changes.

This uniform shift reflected the emergence of new preparatory activity patterns that were not used before learning. These new patterns were identified using a neural repertoire metric^4^ (Fig. 2e, Extended Data Fig. 2e), which revealed that these changes were specific to learning, compared to control sessions without curl fields or with random pulse perturbation forces that simulated the magnitude of the curl field but did not admit learning (Fig. 2f, g). Moreover, we did not observe a uniform shift or repertoire change during VMR learning (Extended Data Fig. 2f, g) where reassociation was observed, consistent with previous studies^18,20^.

## Uniform shifts index motor memories

**Extended Data Fig. 6 |:**
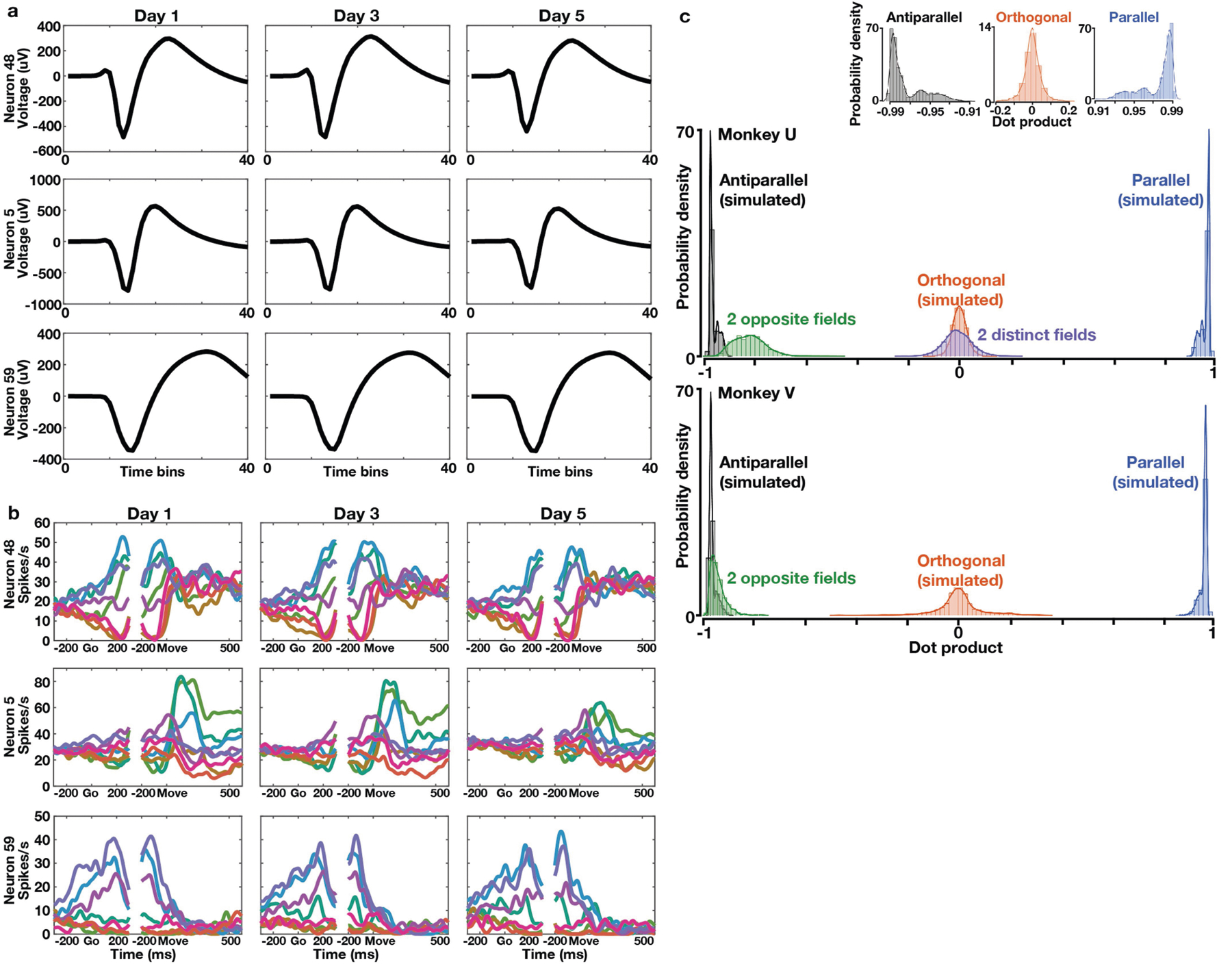
Stability of multi-session recordings and the geometric relationship between uniform-shift axes for learning multiple curl fields. **a, b**, Spike waveforms and peristimulus time histograms (PSTHs) of three example neurons across sessions. The same 71 neurons from monkey U Utah-array recordings were selected post-hoc by comparing waveform correlations and tracked over five successive sessions. **a**, All selected neurons had nearly-identical waveforms. **b**, Like the three example neurons, most selected neurons had similar direction-tuning properties for before-learning reaches across sessions. Go, go cue. Move, movement onset. **c**, Distribution of dot products between uniform-shift axes for learning two opposite fields applied at one reach target sequentially (green) or two distinct fields applied at different reach targets sequentially (purple). We compared them with simulated distributions of dot products between uniform shifts predicted by ‘orthogonal’ (red, around 0), ‘parallel’ (blue, around 1) and ‘antiparallel’ (black, around -1) relationships (see Measurement of geometric relationships between uniform-shift axes in Methods). Top inset, the zoom-in view of each simulated distribution.

**Fig. 3 |:**
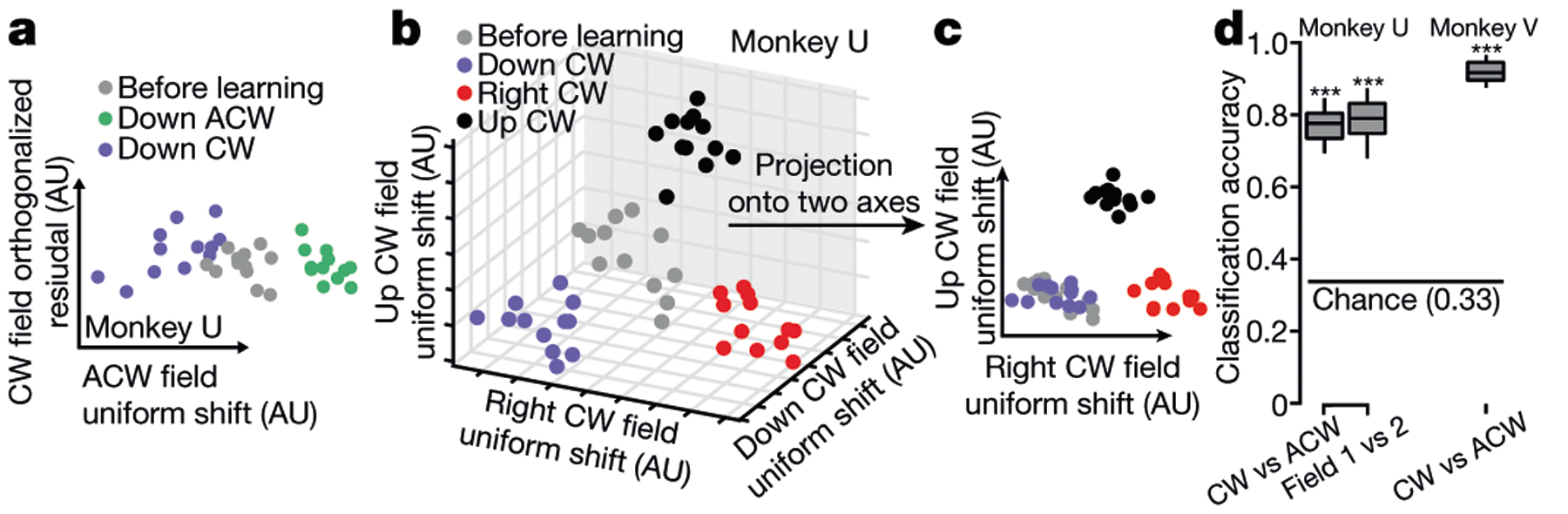
Field-specific geometry of preparatory uniform shifts. **a**, Preparatory states shifted in opposite directions for learning two opposing curl fields at the same trained target sequentially. **b**, Uniform shifts proceeded along nearly orthogonal directions for curl fields at three trained targets 90° apart. **c**, Projection of neural states in **b** onto two axes. **d**, Cross-validated classification accuracy for predicting learning opposite fields (clockwise versus anticlockwise versus before learning) and learning distinct fields (field 1 versus field 2 versus before learning) from single-trial preparatory states, using a minimum distance decoder. ***P < 0.001. See Supplementary Table 1 for statistics.

Next, we tested whether distinct uniform shifts might accompany learning multiple curl fields. We trained monkeys to learn different curl fields sequentially within the same session or over multiple sessions. To compare learning over multiple sessions, we tracked a stable neural population with highly similar cross-session waveforms (Extended Data Fig. 6a, b). We then identified the uniform-shift axis for each learned field and computed the dot product between each pair of axes. For two opposite curl fields applied to the same target, the uniform-shift axes were nearly antiparallel, such that preparatory neural states shifted in opposite directions with respect to the before-learning states (Fig. 3a, Extended Data Fig. 6c). For curl fields trained at different reach directions (up, right or down), the uniform-shift axes were nearly orthogonal (Fig. 3b, c, Extended Data Fig. 6c). Curl-field identity was reliably decoded above chance on the basis of the shifted post-learning preparatory states (Fig. 3d). These geometric relationships suggest that uniform shifts index specific curl fields, mapping motor memories to distinct, precisely arranged locations in neural state space.

## Uniform-shift geometry and interference

**Extended Data Fig. 7 |:**
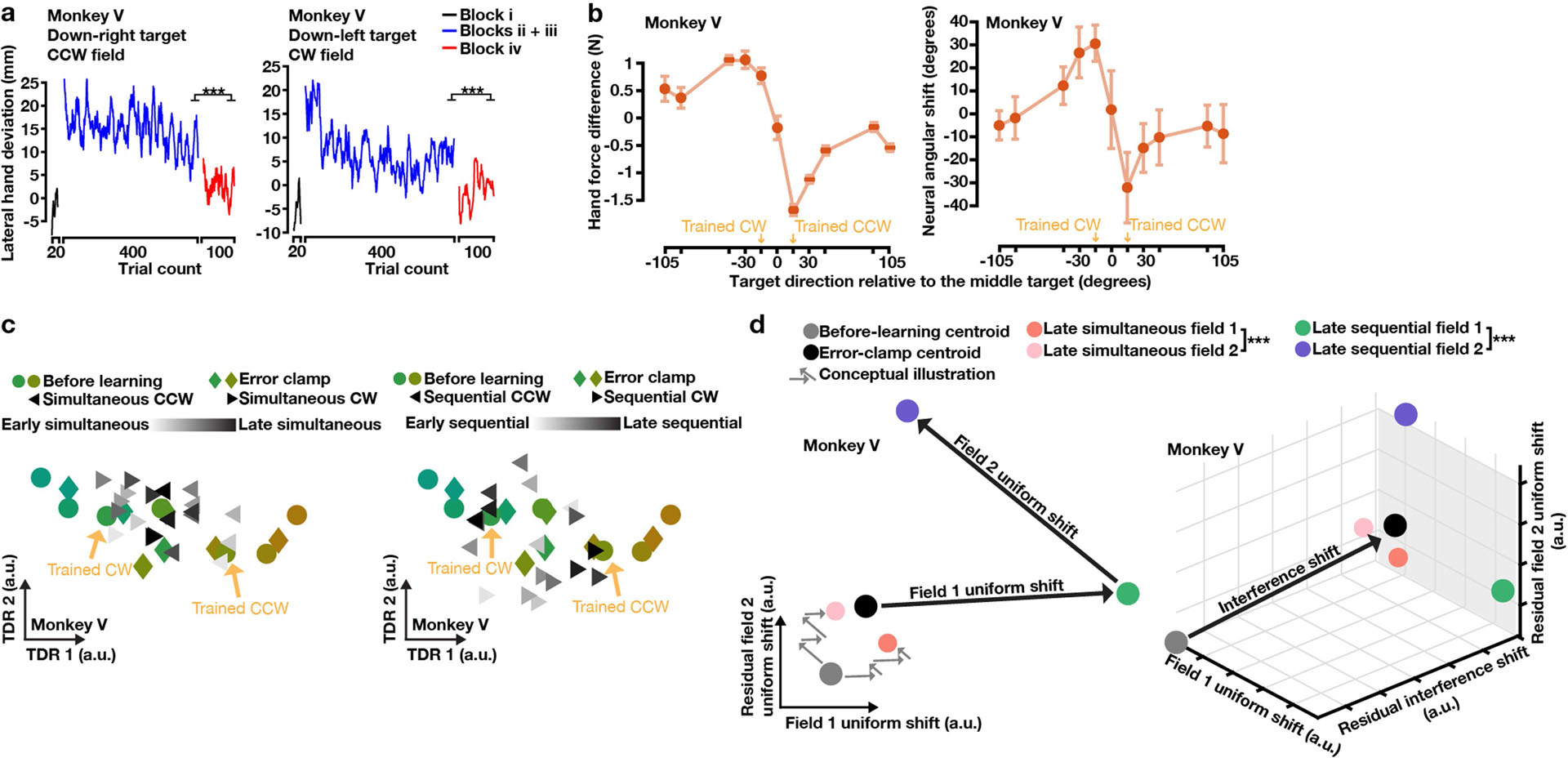
Interference and sequential learning of opposite curl fields applied at two targets 30 degrees apart, monkey V results. **a**, Behavioural learning quantified by lateral hand deviation. Lateral hand deviation in both curl fields decreased slowly during simultaneous learning (blue) and further reduced during sequential learning (red). One-sided Wilcoxon rank-sum test: *P*_*CCW*_ = 1.30 × 10^−10^, *P*_*CW*_ = 1.51 × 10^−11^. **b**, Behavioural and neural generalization of simultaneously learning two fields. Behavioural generalization was measured by perpendicular hand force differences between error-clamp and before-learning trials, and neural generalization was measured by the rotatory angle between before-learning and error-clamp neural states. Zero degree on the x axis, the middle target between the two trained targets. Error bars, s.e.m. from resampling (100 repeats). **c**, Preparatory neural states in the force-predictive TDR subspace. Before-learning states (circles) spatially organized corresponding to reach directions. Error-clamp states (diamonds) rotated counterclockwise for reach targets near the CW field and clockwise for targets near the CCW field. Preparatory states of the two trained targets (triangles) rotated opposite their curl field directions in blocks ii and iii (left panel), and further separated in the sequential-learning block (right panel). Small yellow arrows point to the before-learning states of trained targets. Neural states of seven nearest targets are visualized in c, and quantified neural changes for all 11 targets are shown in b. **d, Left panel:** preparatory activity projected into the subspace spanned by the two field-specific uniform shifts. Without orthogonalization, these two uniform shifts were 128 degrees apart. The uniform shifts were orthonormalized before projection such that: x axis = field 1 uniform shift, y axis = field 2 uniform shift – the projection of field 2 uniform shift on field 1 uniform shift. During simultaneous learning (blocks ii and iii), preparatory states of each field (orange and pink) moved in its specific uniform-shift direction while also progressing in the other uniform-shift direction, and were significantly separated (Hotelling’s T^2^ test: *P* = 2.58 × 10^−6^). Grey arrows illustrate the hypothesized trial-by-trial progression of preparatory states for both fields during simultaneous learning. During sequential learning, preparatory states of each field (green and purple) further separated (Hotelling’s T^2^ test: *P* = 0). **Right panel:** A residual interference shift orthogonal to the field-specific uniform shifts occurred during simultaneous learning. Neural states in the left panel are the projection of neural states into the grey plane in the right panel.

**Fig. 4 |:**
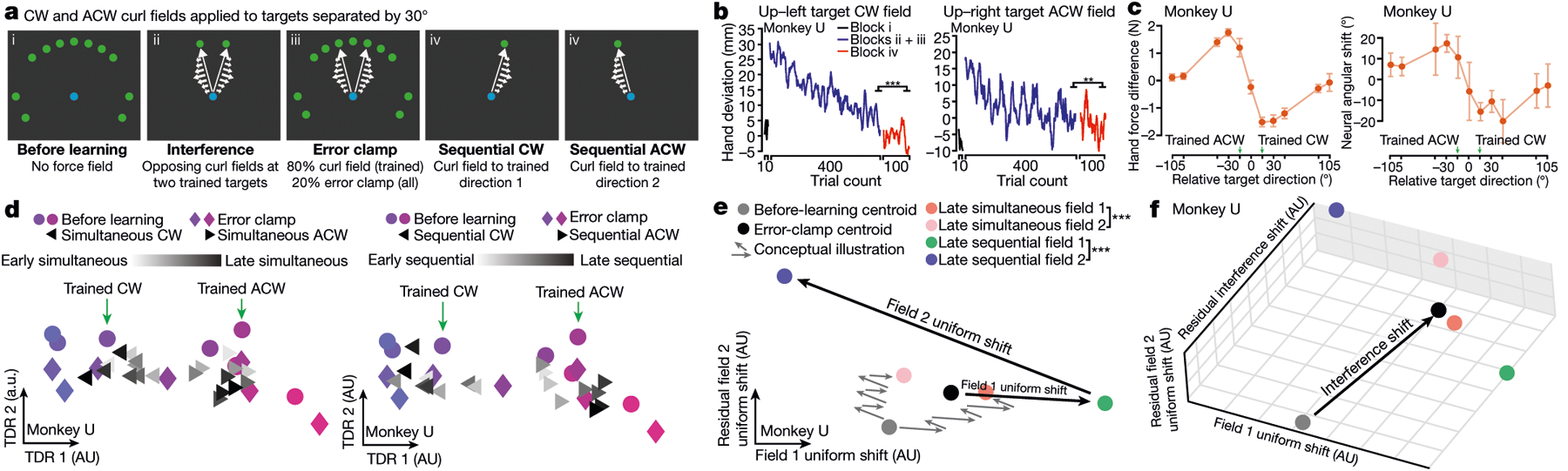
Interference during learning of opposing curl fields at two targets 30° apart. **a**, Task schematic. Opposing fields were trained simultaneously at targets 30° apart (ii–iii), and generalization was probed by error clamp (iii). Finally, each field was trained sequentially (iv). **b**, Hand lateral deviation decreased slowly during simultaneous learning (blue) and reduced further during sequential learning (red). **c**, Behavioural and neural generalization, as in Figs. 1d, 2c. Data are mean ± s.e.m. from resampling. **d**, In the force-predictive TDR subspace, preparatory states for the trained targets (triangles) rotated opposite their curl field directions during simultaneous learning (left) and further separated during sequential learning (right). Preparatory states for seven nearest targets are shown. **e**, Uniform-shift axes for the two fields formed an obtuse angle and partially opposed each other. Grey arrows illustrate the hypothesized trial-by-trial progression of preparatory states for both fields during simultaneous learning (ending with orange and pink states). During sequential learning, preparatory states for both fields (green and purple) further separated. **f**, A residual interference shift orthogonal to the field-specific uniform shifts occurred during simultaneous learning. ***P < 0.001. See Supplementary Table 1 for statistics.

**Fig. 5 |:**
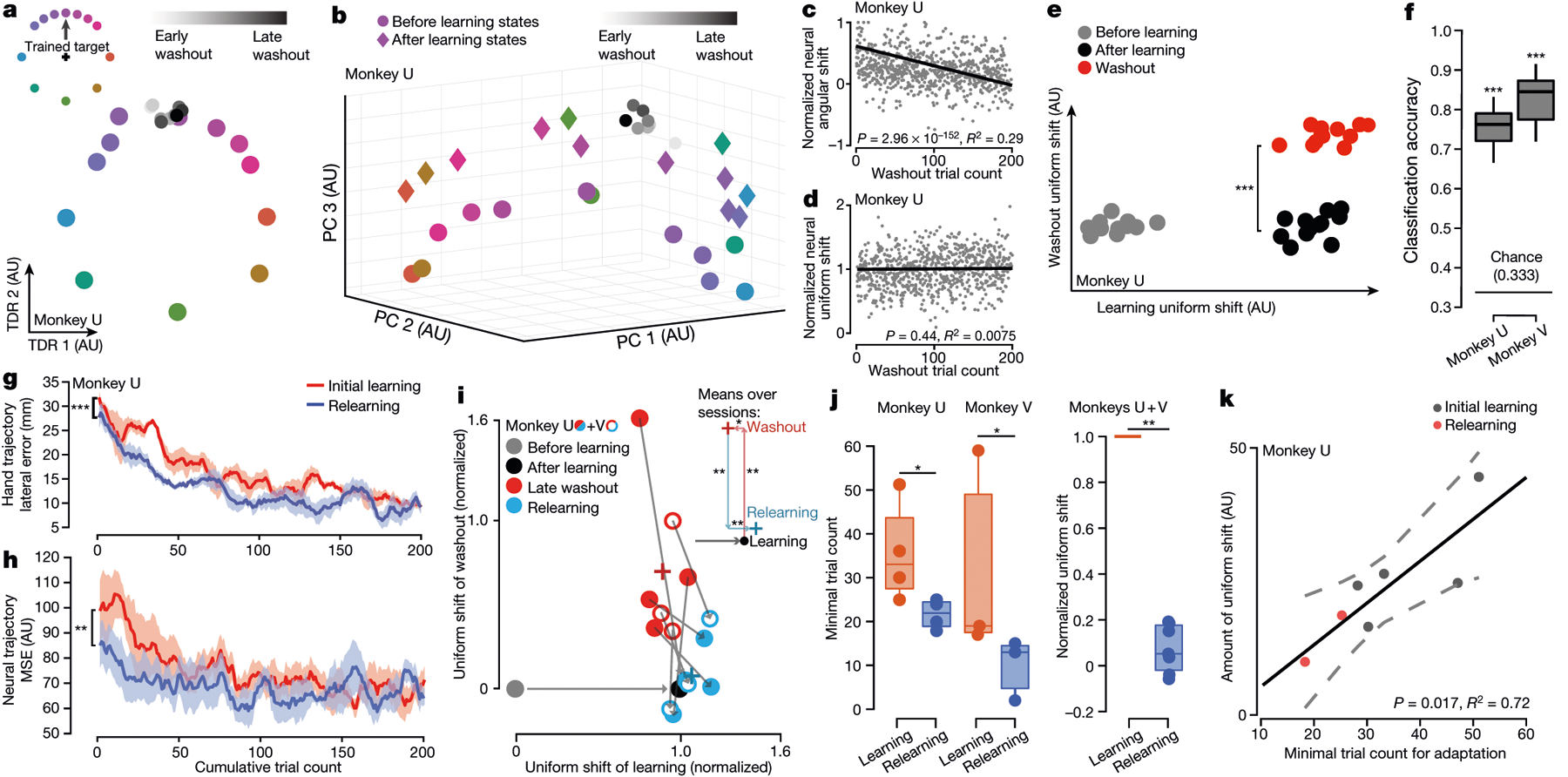
Uniform-shift progression correlates with motor memory retention. **a**, **c**, In the force-predictive TDR subspace, preparatory states for the trained target gradually reverted to the before-learning state during washout. One example session visualized in **a** and four sessions quantified in **c**. **b**, **d**, Preparatory washout states remained separated from before-learning states along the uniform-shift axis. One example session visualized in **b** and four sessions quantified in **d**. **e**, Preparatory washout states shifted uniformly along a dimension orthogonal to the uniform-shift learning axis. **f**, Cross-validated accuracy for classifying before-learning, late-learning and late-washout trials from single-trial preparatory states. **g**, Behavioural relearning progressed faster than initial learning. **h**, Neural trajectories approached late-learning trajectories faster during relearning than initial learning. MSE, mean squared error. In **g**, **h**, shaded area shows s.e.m. across sessions. **i**, Centroids of late-washout and relearning states from individual sessions projected onto normalized uniform-shift learning and washout axes. Inset shows means across seven sessions. A significant shift along the washout axis and a small but significant regression along the learning axis accompanied washout, and reversed during relearning. **j**, Minimum trial count required for behavioural adaptation during relearning was significantly smaller than for initial learning. Normalized progression along the uniform-shift learning axis was significantly smaller through relearning. **k**, The magnitude of preparatory uniform shift correlated strongly with the number of trials required for adaptation. *P < 0.05, **P < 0.01, ***P < 0.001. See Supplementary Table 1 for statistics.

This contrast between orthogonal uniform shifts at well-spaced trained targets and opposing shifts at the same target suggests a connection between uniform-shift geometry and interference. Interference is a slowing of learning that can occur when adapting to opposing fields simultaneously^3,7,8,10^. When two fields interfere, the associated uniform shifts might be oriented so that trial-by-trial changes partially oppose each other. To test this, we designed an interference experiment which interleaved reaches to two targets separated by 30° (within the effect of spatial generalization, Fig. 1d) with opposite curl fields applied (Fig. 4a). Hand deviation errors decreased over hundreds of trials (Fig. 4b, Extended Data Fig. 7a); however, more trials were required to learn either field (more than 400 trials) versus when learning each field individually (fewer than 200 trials; Fig. 5g), indicating that simultaneous learning was slowed by partial interference. In a subsequent sequential-learning block, both fields were trained separately and hand deviation errors further decreased (Fig. 4b, Extended Data Fig. 7a).

Within a force-predictive TDR subspace, preparatory states for the two trained targets gradually rotated in opposite directions during simultaneous learning, and this progression continued during sequential learning, mirroring behavioural performance (Fig. 4d, Extended Data Fig. 7c). At nearby targets, we observed behavioural and neural generalization from learning both fields (Fig. 4c, Extended Data Fig. 7b). We then identified uniform-shift axes for the two fields using preparatory states during sequential learning, which were oriented 127° (monkey U) and 128° (monkey V) apart. This angle is intermediate between orthogonal uniform-shift axes for fields applied at targets 90° apart (Fig. 3b), and antiparallel axes for opposing fields applied at the same target (Fig. 3a). During simultaneous learning, shifts of preparatory states along these axes partially opposed each other, suggesting a neural mechanism of interference (Fig. 4e, Extended Data Fig. 7d).

**Extended Data Fig. 8 |:**
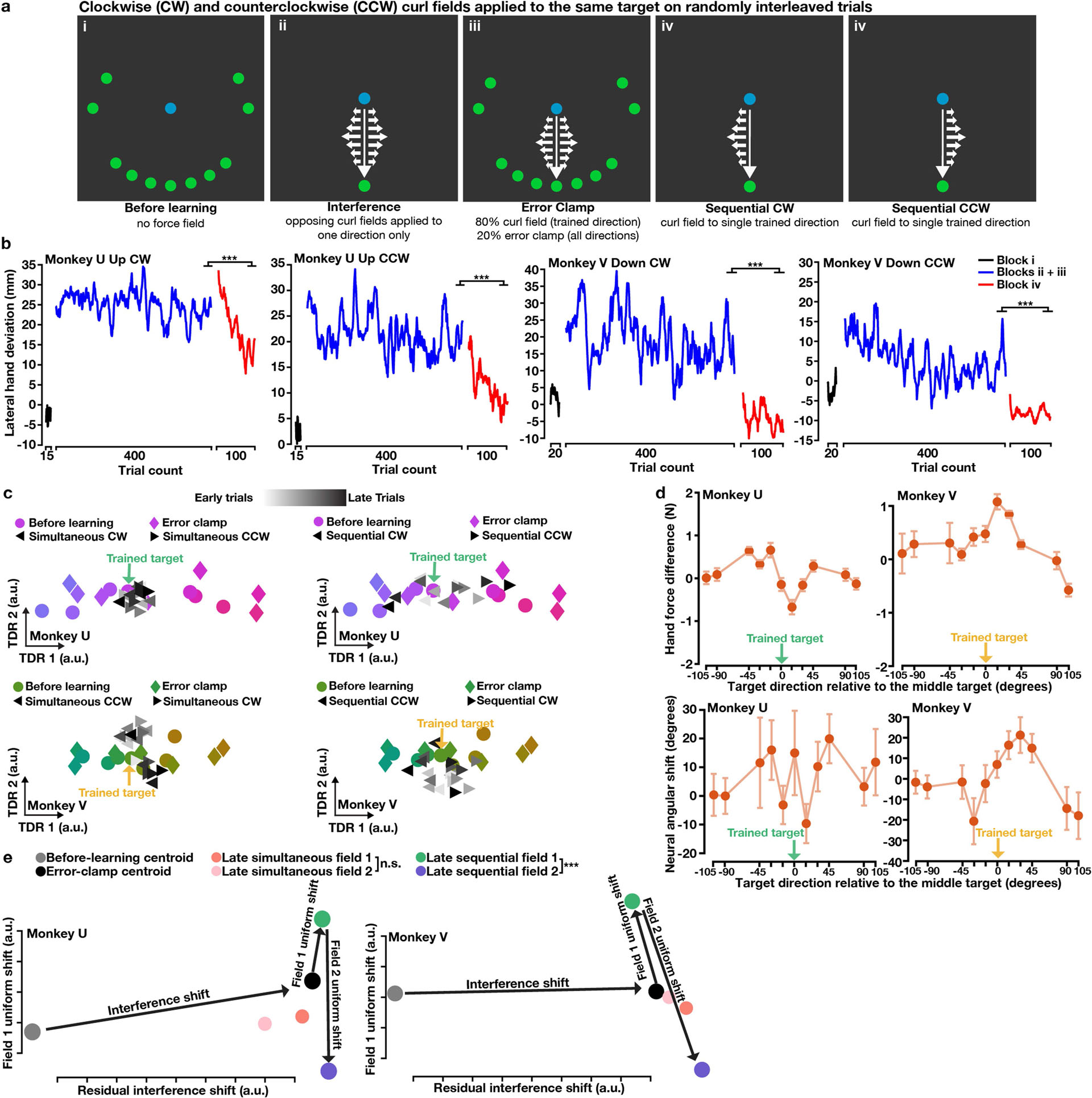
Interference and sequential learning of opposite curl fields applied to the same target. **a**, Block design of the interference experiment. Same as in Fig. 4a except that two opposite curl fields were applied to the same reach target. **b**, Behavioural learning quantified by lateral hand deviation. Hand lateral deviation in both curl fields only slightly decreased during simultaneous learning (blue), and significantly reduced during sequential learning (red). One-sided rank-sum test: monkey U, ***P = 1.51 × 10^−11^; monkey V, ***P = 1.51 × 10^−11^. **c**, Preparatory neural states in the force-predictive TDR subspace. In blocks ii and iii (left panel), preparatory states of the two curl fields (triangles) were mixed together around the before-learning state (circle). Error-clamp states (diamonds) of most targets shifted from their corresponding before-learning states. These shifts did not show coherent patterns across targets or monkeys and were likely unrelated to learning. In the sequential-learning block (right panel), preparatory states of the two curl fields (triangles) gradually rotated opposite their curl field directions. The small arrow points to the before-learning state of the reach target that later had curl fields (trained target). Neural states for seven nearest targets are shown. **d**, Perpendicular hand force differences between error-clamp and before-learning trials (top panel), and the rotatory angle from before-learning to error-clamp neural states (bottom panel), did not show coherent patterns across targets or monkeys. Zero degree on the x axis, the trained target. Error bars, s.e.m. from resampling (100 repeats). **e**, Uniform shifts for learning two curl fields and the residual interference shift were defined in the same way as in Fig. 4e, f. The two field-specific uniform shifts were close to antiparallel (monkey U, dot product = − 0.79; monkey V, dot product = −0.64), and so we could visualize preparatory neural states in a 2D plane spanned by the field 1 uniform shift and the residual interference shift. In blocks ii and iii, preparatory states of the two curl fields (orange and pink) shifted away from the before-learning centroid (grey circle) along the residual interference axis, but they remained close to each other (Hotelling’s T^2^ test: monkey U, *P* = 0.66; monkey V, *P* = 0.98). During sequential learning, preparatory states of the two curl fields (green and purple) were separated by opposite uniform shifts (Hotelling’s T^2^ test: monkey U, *P* = 2.49 × 10^−4^; monkey V, *P* = 2.90 × 10^−5^). **b–e**, One session per monkey. Though just one session of this interference experiment was performed with each monkey, the results were consistent across monkeys and complimentary to findings when monkeys learned multiple fields sequentially, which supported the indexing hypothesis (Fig. 3).

We conducted a second interference experiment with opposite fields applied at the same reach target on randomly interleaved trials (Extended Data Fig. 8a). As expected, monkeys were unable to learn the two fields simultaneously, indicating complete interference^7^, but subsequently learned both fields sequentially (Extended Data Fig. 8b). As the uniform-shift axes associated with opposing fields at a single target were antiparallel (Fig. 3a), we predicted little net progress along this shared dimension during simultaneous learning. Indeed, preparatory neural states for the two fields remained unseparated within the force-predictive subspace and along the shared uniform-shift axis (Extended Data Fig. 8c, e). Subsequently, preparatory states shifted along the antiparallel uniform-shift axes during sequential learning (Extended Data Fig. 8e). Collectively, these findings indicate a correspondence between interference and the geometry of uniform-shift axes during motor preparation.

Additionally, in both interference experiments, we observed a residual neural shift that accompanied interference during simultaneous learning, orthogonal to the field-specific uniform shifts during sequential learning (Fig. 4f, Extended Data Figs. 7d, 8e). Notably, the residual interference shift occurred even when no net learning was observed. We speculate that this residual shift probably relates to an attempt to index neural activity patterns specific to the interference context, which might facilitate strategies tailored to adapting to an unpredictable environment (for example, impedance control to stabilize the limb^32,33^).

## Uniform shift may retain a motor memory

**Extended Data Fig. 9 |:**
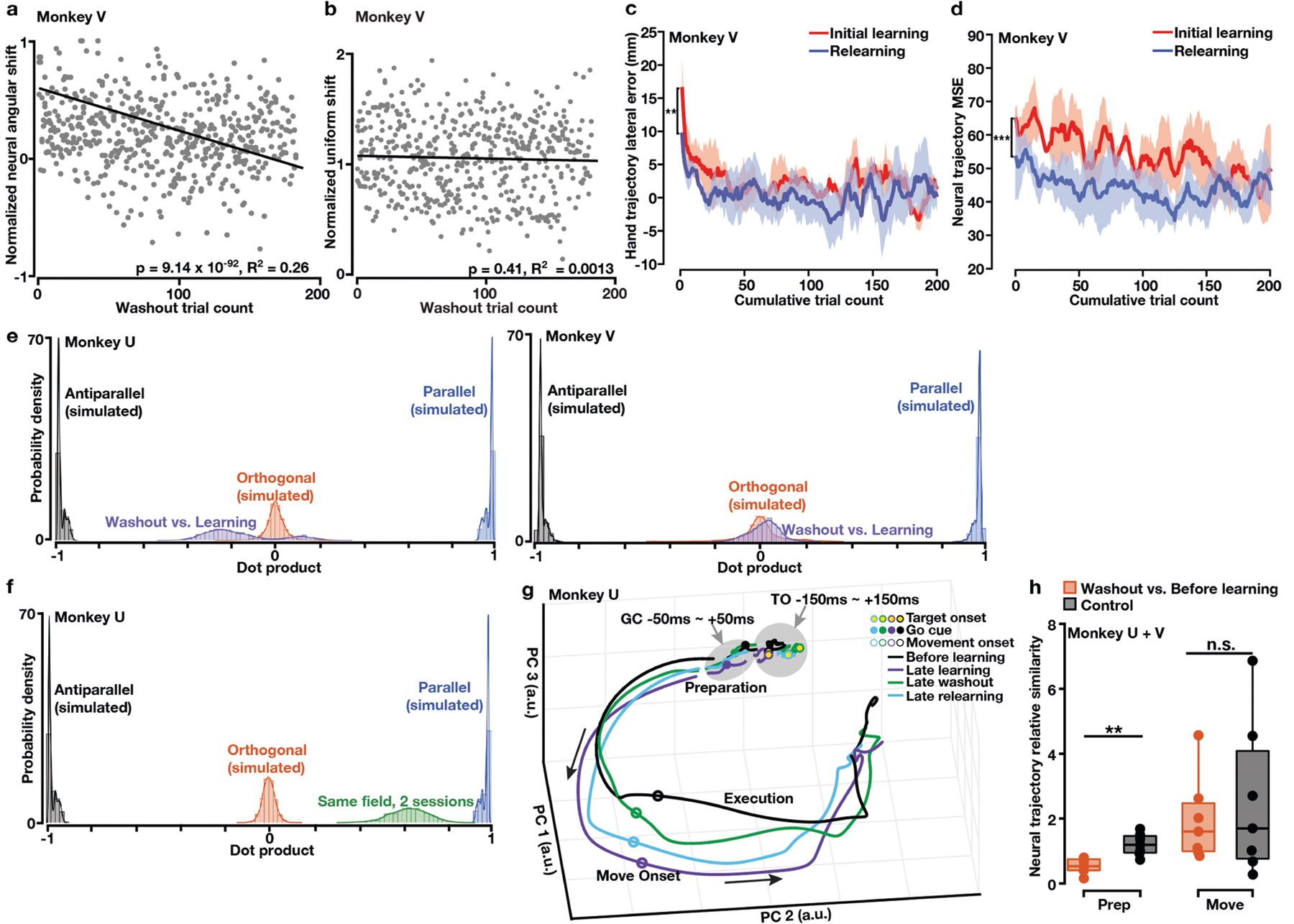
Monkey V washout results and additional information on the neural population correlate of motor memory retention. **a**, The angular difference between washout states and the before-learning state gradually decreased on a single-trial basis (grey dots: single-trial data points from all sessions; solid line: linear regression). Normalized against the maximum in each session. **b**, Distance between washout states and the before-learning state along the uniform-shift learning axis did not show a significant trend of increase or decrease (solid line: linear regression). Each dot is a single trial. **c**, Hand deviation was smaller during relearning than during initial learning (one-sided Wilcoxon rank-sum test: *P* = 0.0015). **d**, Neural trajectories approached late-learning trajectories faster during relearning than initial learning. One-sided rank-sum test: *P* = 6.18 × 10^−4^. **c, d**, Shaded area, s.e.m. across sessions (n = 3). **e**, Distribution of dot products between uniform-shift learning and washout axes (purple), compared to simulated distributions of dot products between uniform-shift axes predicted by orthogonal (red), parallel (blue) and antiparallel (black) relationships. **f**, Distribution of dot products between uniform-shift axes for learning the same curl field in two sessions 18 days apart (green, close to 1). **g**, Neural trajectories of before-learning, late-learning, late-washout and late-relearning conditions (−150 to +150 ms from target onset, covered by the grey circle; −50 to +50 ms from the go cue, covered by the grey ellipse; and −200 to +400 ms from movement onset). Movement preparation and execution periods are noted on the trajectories. Black arrows show the direction of neural trajectories. The late-washout trajectory (green) was less similar to the before-learning trajectory (black) during movement preparation than execution. TO: target onset. GC: go cue. One example session. **h**, During preparatory period (prep), the similarity between late-washout and before-learning neural trajectories was significantly lower than the similarity between before-learning neural trajectories. During movement period (move), the similarity between late-washout and before-learning neural trajectories could compare to the similarity between before-learning neural trajectories. One-sided rank-sum test: seven sessions from monkeys U + V, **P = 0.0012, n.s. *P* = 0.50. For each box, the central line indicates the median, the bottom and top edges indicate the 25th and 75th percentiles of the data, and the whiskers extend to the 5th and 95th percentiles of the data.

Finally, we examined whether learning-induced shifts in preparatory activity persisted after de-adaptation as a motor memory^5,24,34^. Over hundreds of washout trials without the field, monkeys gradually reverted to their before-learning reaching behaviour (Fig. 1b, c, Extended Data Fig. 4a). Washout preparatory states in the force-predictive subspace correspondingly rotated back towards the before-learning states (Fig. 5a, c, Extended Data Fig. 9a). By contrast, along the uniform-shift axis, washout states remained separated from before-learning states (Fig. 5b, d, Extended Data Fig. 9b). Furthermore, preparatory states shifted uniformly again during washout along a second, nearly orthogonal dimension (Fig. 5e, Extended Data Fig. 9e). Before-learning, late-learning, and late-washout conditions could be reliably decoded from single-trial preparatory states (Fig. 5f). Collectively, these results underscore that washout is not simply the reverse of learning and suggest that the persistent uniform shift of preparatory activity potentially retains a motor memory of the learned field.

Furthermore, we performed a relearning experiment in which monkeys were exposed to the same field again after washout within the same session^10^. Monkeys relearned the curl field faster than the initial learning, a hallmark of motor memory retention (Fig. 5g, Extended Data Fig. 9c). Neural trajectories during relearning approached the late-learning neural trajectory faster than during initial learning (Fig. 5h, Extended Data Fig. 9d). Moreover, preparatory states after relearning were indistinguishable from the initial learning states within each session (Fig. 5i). We also observed that uniform shifts for the same field in two sessions 18 days apart were close to parallel (Extended Data Fig. 9f). These results support the hypothesis that the uniform shift indexes and stores a field-specific motor memory.

We also assessed the relationship between distances neural states progressed along the uniform-shift learning axis and behavioural learning rates. Within a session, uniform-shift distances were significantly smaller during relearning than during initial learning (Fig. 5j). Across five sessions with a consistent neural population, uniform-shift distances were strongly correlated with behavioural learning rates (Fig. 5k), suggesting that if preparatory states begin further along a given uniform-shift axis, learning will proceed faster.

## Uniform shift is specific to motor preparation

**Extended Data Fig. 10 |:**
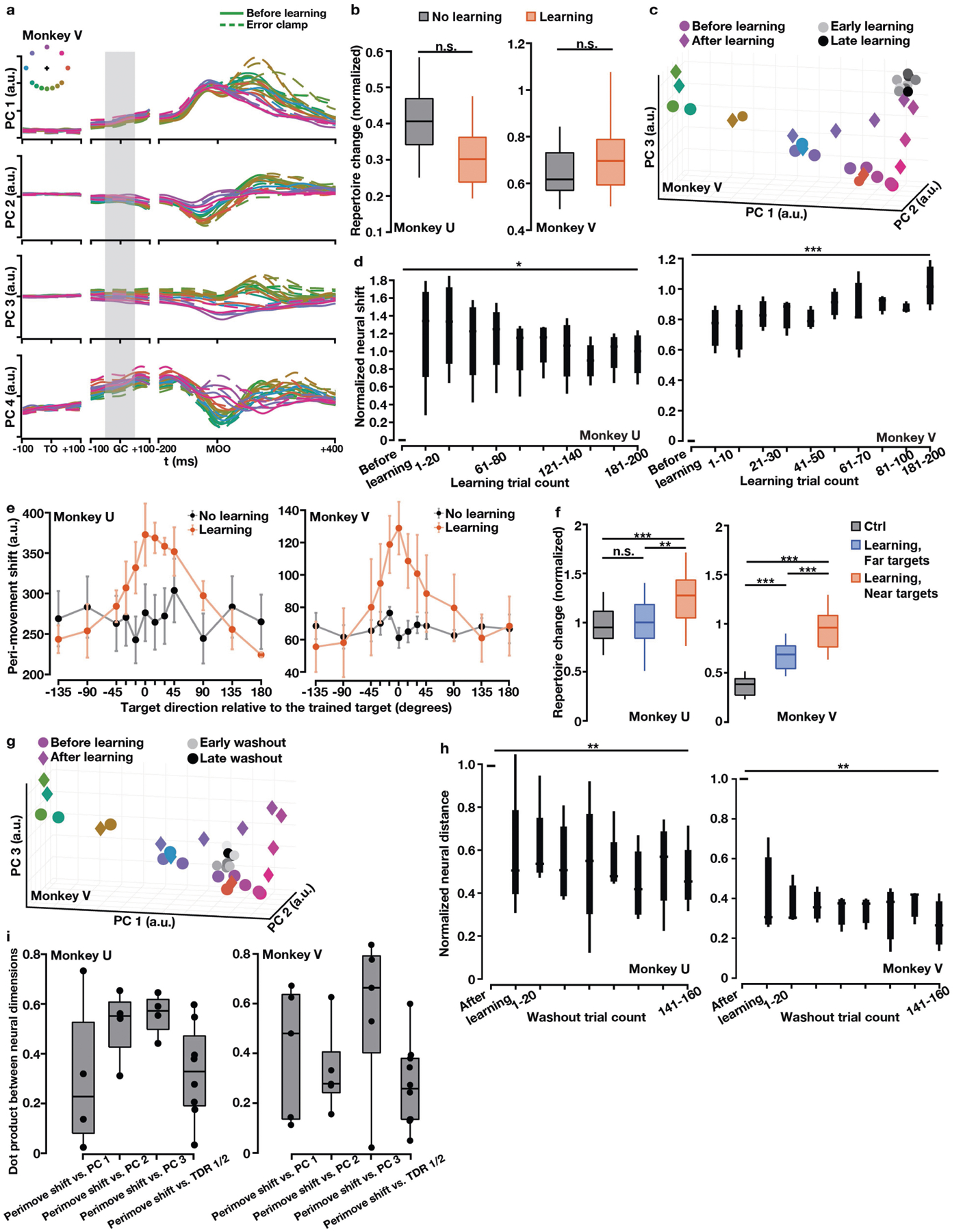
Neural population activity patterns in other time windows. **a**, PCs 1–4 during −100 to +100 ms from target onset (TO), −100 to +100 ms from go cue (GC), and −200 to +400 ms from movement onset (MOO). We applied PCA to trial-averaged neural activity for different reach directions in the before-learning and error-clamp blocks. Across all learning sessions in both monkeys, PC 1 explained 30 – 40% of the total variance, PC 2 explained 10 – 20%, PC 3 explained 8 – 10%, and PC 4 explained 6 – 8%. Neural trajectories in PCs 1–4 were bundled together around the target onset time window and diverged around the go cue time window (preparatory period). Error-clamp neural trajectories all shifted from their corresponding before-learning trajectories in PC 3. The time window −50 to +50 ms from go cue (grey shadow) we chose for preparatory neural state analysis was within the preparatory period and had stronger neural tuning than earlier time windows (e.g., the first 100 ms after target onset). Top left inset: color-coded reach directions. **b**, No significant neural repertoire change of baseline neural activity (before target onset on each trial) after learning the curl field. Black: no-learning control results (n = 36, 36). Red: learning results (n = 48, 36). One-sided Wilcoxon rank-sum test: monkey U, *P* = 0.999; monkey V, *P* = 0.595. **c**–**i**, Patterns of peri-movement neural population states. **c**, Peri-movement states of before-learning (color circles), learning (grey circles) and after-learning error-clamp (diamonds) reaches projected to PCs 1–3. After-learning states of the trained target and its nearby untrained targets left the before-learning states. One example session. **d**, Quantification of neural shift during learning along the ‘peri-movement shift axis’ that connected the before-learning and error-clamp states of the trained target, normalized against the distance between these two states. n = 4 (monkey U), 5 (monkey V). Cuzick’s test: monkey U, *P* = 0.032; monkey V, *P* = 3.92 × 10^−5^. **e**, The Euclidian distance between before-learning and after-learning peri-movement states showed bell-shaped local generalization. Error bars, s.e.m. across sessions (monkey U: n = 4, 3; monkey V: n = 5, 3). **f**, Peri-movement activity patterns showed significantly greater repertoire change for the trained target and near targets than far targets (monkey U: n = 28, 20; monkey V: n = 21, 15). Black: no-learning control sessions (n = 36 for both monkeys). One-sided Wilcoxon rank-sum test: monkey U, *P*_*black vs. blue*_ = 0.26, *P*_*black vs. red*_ = 4.52 × 10^−6^, *P*_*blue vs. red*_ = 0.002; monkey V, *P*_*black vs. blue*_ = 3.70 × 10^−7^, *P*_*black vs. red*_ = 6.02 × 10^−9^, *P*_*blue vs. red*_ = 5.29 × 10^−4^. **g**, Peri-movement states in the same PCA subspace during washout. **h**, Distance between washout and before-learning states decreased significantly along the peri-movement shift axis. Normalized against the distance between the before-learning and after-learning states of the trained target. n = 4 (monkey U), 5 (monkey V). Cuzick’s test: monkey U, *P* = 0.0077; monkey V, *P* = 0.0028. **i**, Pairwise dot products between peri-movement neural dimensions. PCs 1–3 significantly overlapped with the peri-movement shift (one-sided signed-rank test compared to 0: monkeys U + V, n = 9 and *P* = 0.002 for all comparisons). TDR 1 / 2 axes also significantly overlapped with the peri-movement shift (one-sided signed-rank test compared to 0: monkeys U + V, n = 18 and *P* = 1.07 × 10^−4^). For all the box plots, the central line indicates the median, the bottom and top edges indicate the 25th and 75th percentiles of the data, and the whiskers extend to the 5th and 95th percentiles of the data.

The uniform shift emerged during motor preparation and our results were largely insensitive to the preparatory time window analysed (not shown) owing to relatively stationary neural activity during the preparatory period (Extended Data Fig. 10a). By contrast, we did not find repertoire changes in baseline activity (Extended Data Fig. 10b) or uniform shifts in peri-movement activity. Shifts of peri-movement states during learning were local and matched the profile of behavioural generalization (Extended Data Fig. 10c–f). Peri-movement states reverted to before-learning patterns after washout (Extended Data Fig. 10g, h), mirroring the de-adapted movement. Correspondingly, late-washout neural trajectories were more similar to before-learning neural trajectories during the peri-movement period than the preparatory period (Extended Data Fig. 9g, h). Taken together, the uniform shift was a learning-related feature of neural population activity specific to motor preparation.

## Discussion

Through the lens of curl field learning, we identified structured changes in cortical preparatory activity that reflected distinct components of motor learning. We found reassociation-like changes in preparatory activity in a movement-predictive neural subspace closely coupled to changes in movement parameters, similar to those reported in VMR and short-term BCI learning^4,18,26,27,29,30^ (see Supplementary Discussion). Notably, we discovered a shift of preparatory states along an orthogonal neural dimension that occurred uniformly for all reach targets, including those with unaltered movement. In a series of learning experiments with multiple curl fields, we established that these uniform shifts were arranged in neural state space with a precise geometry that appeared to index distinct motor memories and reduce interference. Following washout, the uniform shift persisted even as reaching behaviour de-adapted. This persistent uniform shift correlated with faster relearning and may serve to retain a short-term memory of recent learning.

Cortical preparatory states provide the initial condition of the dynamical system whose evolution generates activity patterns that drive movement^14,21,23,35–37^. Uniform shifts that separate these initial states may serve to isolate learning-induced modifications to the subsequent neural dynamics, thus separating motor memories to facilitate adaptive behavioural improvements in a specific context^7,8^. Conversely, when the motor system is unable to engage separate indices, such as when opposing curl fields are randomly interleaved^3,13^, opposing modifications to neural dynamics adjust the neural trajectory originating from the shared preparatory state, resulting in interference. Recent behavioural studies demonstrate that when certain contextual cues or movement components are added to differentiate movements, opposing fields can be learned without interference^7,8^. Our results suggest that the motor system leverages the shifts of preparatory states along orthogonal neural dimensions to index distinct motor memories, consistent with the central role of movement preparation reported in these studies.

## Extended Data

## Supplementary Material

Supp Mats

Supplementary Table 1

Supplementary Table 2

## Data Availability

The data that support the findings of the current study are available from the corresponding authors upon reasonable request. Source data are provided with this paper.
