# Supplementary material for: Cortical preparatory activity indexes learned motor memories
Source: Nature. Author manuscript; Available in PMC 2023 Jan 19. (PMC9851374; doi:10.1038/s41586-021-04329-x)
Supplement: Supp Mats [file NIHMS1857847-supplement-Supp_Mats.pdf]

nature portfolio

https://doi.org/10.1038/s41586-021-04329-x

Supplementary information

Cortical preparatory activity indexes learned motor memories

In the format provided by the authors and unedited

Supplementary Information:
Cortical preparatory activity indexes learned motor memories

Xulu Sun[*,1,6,✉], Daniel J. O'Shea[*,2,6,✉], Matthew D. Golub[2,6], Eric M. Trautmann[2,6], Saurabh Vyas[3,6], Stephen I. Ryu[2,4], Krishna V. Shenoy[2,3, 5-7,✉]

1 Department of Biology, Stanford University, Stanford, CA 94305, USA

2 Department of Electrical Engineering, Stanford University, Stanford, CA 94305, USA

3 Department of Bioengineering, Stanford University, Stanford, CA 94305, USA

4 Department of Neurosurgery, Palo Alto Medical Foundation, Palo Alto, CA 94301, USA

5 Department of Neurobiology, Stanford University, Stanford, CA 94305, USA

6 Wu Tsai Neuroscience Institute, Stanford University, Stanford, CA 94305, USA

7 Howard Hughes Medical Institute, Stanford University, Stanford, CA 94305, USA

✉ Correspondence should be addressed to X.S. (`xulu.sun@ucsf.edu`), D.J.O. (`djoshea@stanford.edu`), and K.V.S. (`shenoy@stanford.edu`)

***** These authors contributed equally.

This document contains:

- Supplemental Note 1: Changes of initial conditions vs. changes of the neural dynamics matrix
- Supplemental Note 2: Alternative explanations for observed uniform shift
- Supplemental Note 3: Geometry of neural dimensions during the peri-movement period
- Methods
- Supplementary Discussion

Supplemental Note 1

Changes of initial conditions vs. changes of the neural dynamics matrix

In this study, we found that a uniform shift separated after-learning preparatory neural states from the before-learning states, which then generated very different neural trajectories for the newly learned movements in the curl field. This agrees with the "initial-condition theory" shown by previous papers (Afshar et al., 2011; Churchland et al., 2012; Vyas, Golub, et al., 2020). Conversely, if the before- and after-learning neural population states were not separated during the preparatory period, recent theoretical work could provide an alternative explanation as to why the results would still be consistent with the dynamical systems framework and literature (Linderman et al. 2016).

The initial-condition hypothesis often assumes (for simplicity) that the neural dynamical system in the motor cortex is autonomous and there is only one dynamics matrix A before and after learning:

$$\dot{\boldsymbol{x}}(t) = \boldsymbol{A}\boldsymbol{x}(t) + \boldsymbol{B}\boldsymbol{u}(t)$$

where $\boldsymbol{x}(t)$ is the (vector) neural state at time t, $\boldsymbol{u}(t)$ is the (vector) input to the dynamical system at time t and $\boldsymbol{B}$ is the input projection matrix. In this case, the initial-condition hypothesis says that if the initial condition (the preparatory neural state, i.e., $\boldsymbol{x}(t = 0)$) is the same, then one expects to see the same neural trajectory, when there are no external inputs $\boldsymbol{u}(t)$. Finding that neural trajectories cross is what is most often meant by high tangling. In contrast, finding that different initial conditions evolve into different neural trajectories is what is meant by low tangling; technically, trajectories should not come close together yet diverge later in an autonomous system (Russo et al., 2018).

However, in reality, the motor cortical dynamics are non-autonomous. Therefore, there could be another scenario during learning where there are two dynamics matrices, $\boldsymbol{A}_{\text{before learning}}$ and $\boldsymbol{A}_{\text{after-learning}}$ such that:

$$\text{Before learning:} \quad \dot{\boldsymbol{x}}(t) = \boldsymbol{A}_{\text{before learning}}\boldsymbol{x}(t) + \boldsymbol{B}\boldsymbol{u}(t) \tag{1}$$

$$\text{After learning:} \quad \dot{\boldsymbol{x}}(t) = \boldsymbol{A}_{\text{after learning}}\boldsymbol{x}(t) + \boldsymbol{B}\boldsymbol{u}(t) \tag{2}$$

With these two dynamics matrices, even if the neural trajectories before- and after-learning were initiated from the same initial condition (preparatory state), they could evolve separately due to different dynamics matrices and have different neural trajectories. Our results that the initial conditions before and after learning were separated at the time of the go cue cannot tell us whether the dynamics matrix $\boldsymbol{A}$ changes after learning, and investigating this would be quite interesting for future work to test if learning changes the initial conditions, or the dynamics matrix, or both.

Supplemental Note 2

Alternative explanations for observed uniform shift

A central result we presented in this manuscript was the uniform shift of neural preparatory states accompanying curl field adaptation to a specific target, and we argued that this uniform shift was both learning specific and motor memory specific. These two findings support the possible role of the uniform shift in indexing distinct motor memories. This line of reasoning leverages the fact that the behavioral changes demanded by adaptation are spatially localized around the trained target(s), whereas the uniform shift translates the preparatory neural states equally for all reaching targets, including those far from the trained target for which no behavioral changes were observed. The validity of this line of reasoning rests on the assumption that the uniform shift results from learning-related changes and not by some other cause that would in some sense "trivialize" the observed uniform shift. In this note, we consider a set of possible alternative explanations for the observed uniform shift in preparatory activity during curl field learning. We present a combination of methodological, analytical, and empirical evidence to address whether these

proposed alternative explanations could explain the uniform shift we observed in preparatory activity. We found that these alternative explanation did not explain our results.

We begin with an analytical formulation of the uniform shift, and then consider a number of alternative explanations for the uniform shift.

Analytical formulation of uniform shift

Direction tuning We begin by considering the neural preparatory activity states accompanying each target direction, indexed by θ, and whether the reach is before-learning b or adapted a, indexed by $c = \{b, a\}$. We begin by describing the before-learning preparatory firing rate by a cosine-tuned relationship to movement direction:

$$y_n(\theta, b) = \xi_n \cos(\theta - \theta_{pd,n}) + \mu_n$$

Where $y_n(\theta, b)$ is the preparatory firing rate for neuron n on the before-learning condition towards direction θ, ξ_n is the tuning amplitude, $\theta_{pd,n}$ is the preferred direction, and μ_n is the mean firing rate for neuron n. Each of the N neurons' preparatory firing rates are then described by this equation, as each n. By expanding the cosine as a dot product between the target direction vector and the preferred direction vector, this is often rewritten as a linear expression:

$$y_n(\theta, b) = \beta_{x,n} \cos(\theta) + \beta_{y,n} \sin(\theta) + \mu_n \quad (3)$$

Here the $\beta_{x,n}$ and $\beta_{y,n}$ coefficients define the direction tuning preferences of neuron n, forming a 2D vector with magnitude ξ_n and direction $\theta_{pd,n}$.

Endpoint force tuning Naturally, reaching through force fields requires the production of different force profiles, and the firing patterns of neurons in motor cortex reflect these forces, both during movement preparation and execution. Consequently, we primarily consider a similar functional form of preparatory firing rates in terms of forces rather than spatial direction:

$$y_n(\theta, b) = \beta_{x,n} f_x(\theta, b) + \beta_{y,n} f_y(\theta, b) + \mu_n \quad (4)$$

Here, $f_x(\theta, b), f_y(\theta, b)$ are the time-averaged end point forces produced along the x, y directions for a specific before-learning reach condition.

In the manuscript, we used targeted dimensionality reduction (TDR) (Mante et al., 2013) to identify dimensions of neural activity (directions in the N dimensional neural population space). The X and Y force axes identified were defined as the vector of stacked coefficients over neurons, normalized to unit length and orthogonalized relative to each other, i.e. $\boldsymbol{\beta}_x$ and $\boldsymbol{\beta}_y \in \mathbb{R}^N$.

True uniform shift In the after-learning adapted state, we observed that the firing rates were shifted uniformly across conditions in a third dimension. In our model, the adapted firing rates of each neuron can be described by:

$$y_n(\theta, a) = \beta_{x,n} f_x(\theta, a) + \beta_{y,n} f_y(\theta, a) + s_n + \mu_n \quad (5)$$

Here, changes in the firing rate from the before-learning to adapted conditions ($b \to a$) result from two sources. First, from changes in the force production for conditions (particularly near the trained target) where $f_{\{x,y\}}(\theta, b) \neq f_{\{x,y\}}(\theta, a)$. And second, from the uniform shift itself, described by the vector $\boldsymbol{s} \in \mathbb{R}^N$ formed by stacking the s_n over neurons. In the manuscript, we showed that the uniform shift axis $\boldsymbol{u}$ could be described using TDR (Extended Data Fig. 2i). This amounts to stacking the s_n coefficients into vector $\boldsymbol{s} \in \mathbb{R}^N$, orthogonalizing $\boldsymbol{s}$ relative to $\boldsymbol{\beta}_x$ and $\boldsymbol{\beta}_y$ to yield $\boldsymbol{s}_{\text{orth}}$, and then normalizing $\boldsymbol{s}_{\text{orth}}$ to yield $\boldsymbol{u}$. Thus, $\boldsymbol{u}^\mathsf{T}\boldsymbol{\beta}_x = \boldsymbol{u}^\mathsf{T}\boldsymbol{\beta}_y = 0$.

In this formulation, the observed shift in preparatory states along the uniform shift axis u may be calculated by projection:

$$\begin{aligned}\boldsymbol{u}^\top\left[\boldsymbol{y}(\theta,a)-\boldsymbol{y}(\theta,b)\right] &= \boldsymbol{u}^\top\left[\boldsymbol{\beta}_x\left(f_x(\theta,a)-f_x(\theta,b)\right)+\boldsymbol{\beta}_y\left(f_y(\theta,a)-f_y(\theta,b)\right)+\boldsymbol{s}\right]\\ &= \boldsymbol{u}^\top\boldsymbol{\beta}_x\left[f_x(\theta,a)-f_x(\theta,b)\right]+\boldsymbol{u}^\top\boldsymbol{\beta}_y\left[f_y(\theta,a)-f_y(\theta,b)\right]+\boldsymbol{u}^\top\boldsymbol{s} \quad \Big) \ \boldsymbol{u}^\top\boldsymbol{\beta}_x=\boldsymbol{u}^\top\boldsymbol{\beta}_y=0\\ &= \boldsymbol{u}^\top\boldsymbol{s}\\ &= \|\boldsymbol{s}_{\mathrm{orth}}\|\end{aligned}$$

The projection of the preparatory states onto the uniform-shift axis identified via TDR thus isolates the contribution of these uniform shift terms s_n that alter preparatory activity orthogonally to the endpoint force axes.

We note that this projection onto the uniform shift axis naturally does not depend on the target direction, such that the expectation with respect to θ is the same:

$$\mathbb{E}_\theta\left[\boldsymbol{u}^\top\left[\boldsymbol{y}(\theta,a)-\boldsymbol{y}(\theta,b)\right]\right]=\|\boldsymbol{s}_{\mathrm{orth}}\|$$

Lastly, a minor technical point. We note that this definition of the uniform shift axis $\boldsymbol{u}$ differs subtly from the definition used throughout the manuscript. In the manuscript, we defined the uniform shift axis as the vector which connected the centroids of the neural preparatory states across target directions from before learning to after learning, orthogonalized against the TDR force axes $\boldsymbol{\beta}_x$ and $\boldsymbol{\beta}_y$. Here, we construct $\boldsymbol{u}$ directly within the TDR to simplify the derivation. We note that the uniform shift axis estimates are very similar using the two approaches; Extended Data Fig. 2i shows preparatory neural states along the uniform shift vector $\boldsymbol{u}$ in an example session, constructed using the TDR approach used here. Furthermore, none of the analytical derivations below rely on this definition of $\boldsymbol{u}$, instead requiring only that the uniform shift axis be orthogonal to the TDR force axes, so all arguments below hold for the centroid-connecting uniform shift as well.

The alternative explanations we consider below are:

1. Background changes in neuron offset, e.g. background changes in tuning (Rokni et al., 2007)
2. Changes in neuron preferred direction
3. Changes in neural state due to the use of error clamp
4. Changes in neural state due to block switching
5. A change in endpoint forces related to the learned field
6. A change in neuron force preferred direction
7. A change in muscle forces related to the learned field
8. Changes in posture, and specifically posture-related changes in tuning amplitude or gain
9. Changes in hand speed

We note that these alternatives fit broadly into two classes: changes in neural response properties independent of changes in behavior but not learning-specific (1-4) and changes in behavior or motor output (5-9). For five of these alternatives where it is possible to quantitatively estimate their possible contributions, we construct alternative functional forms of $y_n(\theta,a)$ which allow for other kinds of response changes in the adapted conditions, but specifically do not include a uniform shift term. For each of the proposed alternatives, we fit the corresponding model to the recorded preparatory activity, and ask how much of the variance of the observed uniform shift (i.e. the shift in preparatory state along $\boldsymbol{u}$) can be explained. We perform five-fold cross validation and report the cross-validated R^2 in Supplementary Table 2. We find that inclusion of the true uniform shift term as in Eqn. 5 is essential to explain the observed uniform shift.

1 Background drift

Rokni et al., 2007 report background changes of neural tuning curves that occur slowly over many trials during normal reaches in a familiar environment. These background changes occur in tandem with and

on top of learning-related changes accompanying curl field adaptation. Rokni et al., 2007 parcellate these changes into two sources: changes in the tuning curve offset (μ_n in our formulation) and changes in the cosine-tuning (ξ_n and $\theta_{pd,n}$ in our formulation). Here, we discuss the background changes in offset, and discuss the changes in cosine-tuning in the following section. Background changes in offset affect the firing rates observed for all reach directions, as did the uniform shift that we found during curl field learning. Because these changes in offset would use the same functional form as the true-uniform shift in Eqn. 5, it is possible for these changes in offset to create a uniform shift of neural states along an orthogonal axis in neural state space.

However, there are several important differences between our learning-related uniform shift results and the results reported in Rokni et al., 2007. First, we note that Rokni et al., 2007 are reporting changes in the neural tuning during movement and do not study preparatory activity. We are not aware of any reports demonstrating similar background drifts in neural tuning curve offsets during motor preparation, and in particular no reports of drifts in offset that appear specifically during preparation and *not* during movement, which would be required to match the preparation-specific uniform shift we report here. Second, and more critically, Rokni et al., 2007 find that changes in neural tuning in the learning experiment (calculated between before-learning and after-washout trials) are not significantly different from the background changes that they observe in the control experiment with no learning. That is, these background changes are not specifically related to learning. In contrast, the uniform shift we observed occurred only during learning. No uniform shift was observed either in control no-learning sessions of equivalent duration (which matches the non-learning experiments of Rokni et al., 2007; see Fig. 2e, Extended Data Fig. 2e) or in control experiments with randomly delivered mechanical perturbations (which required generation of larger muscles forces in an unfamiliar environment, Fig. 2g). Furthermore, the background changes reported by Rokni et al., 2007 are considered to be the result of drift due to noise that is passively tolerated due to the inherent redundancy of a motor cortex with many neurons and few readout dimensions for behavior (i.e. a very large output-null space). In our results, the uniform shift was specific to parameters of the learned curl field, both in terms of curl field direction and the directional target to which the curl field was trained (Fig. 3). For these reasons, we believe that the continuously occurring, non-specific background drift reported by Rokni and colleagues is fundamentally different from the learning-related uniform shift we observed during curl field adaptation, which we argued served to index distinct motor memories. Furthermore, because no results are presented for motor preparatory activity in Rokni et al., 2007, we were unable to perform further direct, quantitative comparisons.

2 Changes in preferred direction

We now consider changes in neurons' preferred directions from the before-learning to adapted conditions, as have been previously reported, for example in Rokni et al., 2007.

We return to our original formulation of cosine-direction-tuned preparatory firing rates from Eqn. 3, but in the adapted condition, we then allow for each neuron to have a different preferred direction, resulting in new tuning coefficients $\alpha_{x,n}$ and $\alpha_{y,n}$:

$$\begin{aligned} y_n(\theta, b) &= \beta_{x,n} \cos(\theta) + \beta_{y,n} \sin(\theta) + \mu_n \\ y_n(\theta, a) &= \alpha_{x,n} \cos(\theta) + \alpha_{y,n} \sin(\theta) + \mu_n \end{aligned} \tag{6}$$

Technically, since in this formulation, the norm of the vector $[\alpha_{x,n}, \alpha_{y,n}]$ is allowed to differ from that $[\beta_{x,n}, \beta_{y,n}]$, which allows for the neural tuning gain to change due to adaptation as well as the preferred direction. We proceed with this more general model so as to allow for more uniform-shift variance to potentially be explained away both both mechanisms operating in tandem.

If we compute the difference between adapted and before-learning preparatory states, we have:

$$y_n(\theta, a) - y_n(\theta, b) = (\alpha_{x,n} - \beta_{x,n}) \cos(\theta) + (\alpha_{y,n} - \beta_{y,n}) \sin(\theta)$$

At the neural population population level, these changes will result in a rotation of the XY TDR plane. To see this, note that the TDR plane in the before-learning condition would be spanned by the vectors $\boldsymbol{\beta_x}$ and $\boldsymbol{\beta}_y$,

formed by stacking the individual $\beta_{x,n}$ and $\beta_{y,n}$ regression coefficients for each neuron and orthogonalizing with respect to each other. In the adapted conditions, the TDR plane would be instead spanned by the orthogonalized vectors $\boldsymbol{\alpha}_x$ and $\boldsymbol{\alpha}_y$.

If we project this difference onto the uniform shift axis, we have:

$$\begin{aligned}\boldsymbol{u}^\top [\boldsymbol{y}(\theta, a) - \boldsymbol{y}(\theta, b)] &= \boldsymbol{u}^\top [(\boldsymbol{\alpha}_x - \boldsymbol{\beta}_x)\cos(\theta) + (\boldsymbol{\alpha}_y - \boldsymbol{\beta}_y)\sin(\theta)] \\ &= \boldsymbol{u}^\top(\boldsymbol{\alpha}_x - \boldsymbol{\beta}_x)\cos(\theta) + \boldsymbol{u}^\top(\boldsymbol{\alpha}_y - \boldsymbol{\beta}_y)\sin(\theta)\end{aligned}$$

noting that we orthonormalize $\boldsymbol{u}$ off of the $\boldsymbol{\beta}$ vectors fit to force axes as in Eqn. 4, which will not perfectly coincide with coefficients fit to the direction-tuned model of Eqn. 3.

So, if the tuning directions change such that the change in the adapted TDR axes $\boldsymbol{\alpha}_x - \boldsymbol{\beta}_x$ and $\boldsymbol{\alpha}_y - \boldsymbol{\beta}_y$ project into the uniform shift axis $\boldsymbol{u}$, the preparatory states can change along the uniform shift axis as well. However, we observed a consistent shift of all preparatory states (for all directions θ) along the uniform shift axis. If we now marginalize over target direction, we see that changes in preferred directions cannot drive a consistent shift along $\boldsymbol{u}$ that is common to all conditions:

$$\begin{aligned}\mathbb{E}_\theta\left[\boldsymbol{u}^\top [\boldsymbol{y}(\theta, a) - \boldsymbol{y}(\theta, b)]\right] &= \mathbb{E}_\theta\left[\boldsymbol{u}^\top(\boldsymbol{\alpha}_x - \boldsymbol{\beta}_x)\cos(\theta) + \boldsymbol{u}^\top(\boldsymbol{\alpha}_y - \boldsymbol{\beta}_y)\sin(\theta)\right] \\ &= \boldsymbol{u}^\top(\boldsymbol{\alpha}_x - \boldsymbol{\beta}_x)\mathbb{E}_\theta[\cos(\theta)] + \boldsymbol{u}^\top(\boldsymbol{\alpha}_y - \boldsymbol{\beta}_y)\mathbb{E}_\theta[\sin(\theta)] \\ &= 0\end{aligned}$$

Essentially, changes in preferred direction can reorient the preparatory states in neural population space, but they cannot shift the origin around which the preparatory states are radially arranged. Consequently, changes in preferred direction cannot result in the pattern of uniform shifts we observed. Nevertheless, we fit the model in Eqn. 6 allowing for altered preferred directions using regression. We then ask how much of the variance of the observed uniform shift can be explained by changes in preferred direction, reporting the cross-validated R^2 (Supplementary Table 2). This model is fit using a finite set of conditions non-uniformly distributed in direction, so the R^2 need not be 0.

3 Error clamp

We also consider the use of the error clamp is itself responsible for the appearance of the uniform shift. In theory, the error clamp should be largely imperceptible to the subject, as it simply applies forces to match the anticipated forces that the subject applies according to its internal model of the curl field. However, minor deviations in the hand trajectory from a straight line, or practical limitations in the fidelity of the haptic rendering of the error clamp could potentially create the (rather unlikely) situation where these error clamp trials are considered a separate context from the non error-clamp trials, which is then reflected ahead of time during the preparatory activity (before the error clamp is even applied, so necessarily anticipatory) such that this context distinction could create the uniform shift. To address this, we compared the 'error-clamp no-learning' neural state to the 'no-clamp no-learning' state in control no-learning sessions, and found that they were not significantly different (Extended Data Fig. 5a). The late-learning and error-clamp states of the trained reach target were also not significantly different (Extended Data Fig. 5b). Consequently, the use of the error clamp to probe behavioral generalization curves did not itself contribute to the uniform shifts in neural preparatory activity.

As presented in the main results, we observed the reassociation-like neural changes in the force-predictive 2D TDR plane constructed from only before-learning trials. To rule out that this 2D TDR plane reoriented after learning and consequently contributed to the uniform shift, we performed the same TDR regression using only error-clamp trials to build the 2D TDR subspace. We then took the dot products of before-learning and error-clamp TDR axes (Extended Data Fig. 5d). Our results showed that TDR 1 and 2 axes after learning remained aligned with the before-learning TDR axes. The error-clamp TDR 1 and TDR 2 axes were also orthogonal to the uniform-shift axis, similar to the before-learning TDR axes.

4 Block switching

Another possibility is that the uniform shift during the learning block appears simply as an artifact of experimental block switching. We do not think this explanation is viable, because we observed the uniform shift persisting after washout (Fig. 5b, d; Extended Data Fig. 9b) and did not observe the uniform shift during VMR learning (Extended Data Fig. 2f, g), which was conducted using an identical before learning, learning, washout trial block structure. Furthermore, the geometric relationships between different uniform shifts reflected the identity of learned curl fields (Fig. 3), arguing that the uniform shift is specifically learning-related and curl field specific, rather than reflecting an experimental artifact of block switching.

5 Changes in endpoint forces

First, we consider changes in preparatory activity that accompany changes in the movement forces that will subsequently be produced. Because the curl field is trained at only a single target location, the dominant changes in hand forces are concentrated at that target location with a bell-shaped generalization curve. However, we consider the possibility that a change in hand forces common to all targets could result in the appearance of a uniform shift in preparatory activity. To test for this, we consider changes in preparatory activity that result from differences in endpoint forces alone:

$$\begin{aligned} y_n(\theta, b) &= \beta_{x,n} f_x(\theta, b) + \beta_{y,n} f_y(\theta, b) + \mu_n \\ y_n(\theta, a) &= \beta_{x,n} f_x(\theta, a) + \beta_{y,n} f_y(\theta, a) + \mu_n \end{aligned} \tag{7}$$

If we compute the difference between adapted and before-learning preparatory states, and then take an expectation across reach directions,we have:

$$\begin{aligned} \mathbb{E}_\theta \left[y_n(\theta, a) - y_n(\theta, b) \right] &= \beta_{x,n} \mathbb{E}_\theta \left[f_x(\theta, a) - f_x(\theta, b) \right] + \beta_{y,n} \mathbb{E}_\theta \left[f_y(\theta, a) - f_y(\theta, b) \right] \\ &= \beta_{x,n} \Delta f_x + \beta_{y,n} \Delta f_y \end{aligned} \tag{8}$$

where ΔF_x and ΔF_y are the mean changes in hand forces from before-learning to the adapted conditions. If these changes in hand forces are reflected systematically in the neural population, they could result in the preparatory activity shifting uniformly, but in a manner coupled to the behavioral output. However, when we project these differences in firing rates onto the uniform shift axis, they disappear because the the uniform shift axis has been orthogonalized against $\boldsymbol{\beta}_x$ and $\boldsymbol{\beta}_y$.

$$\mathbb{E}_\theta \left[\boldsymbol{u}^\mathsf{T} \left(\boldsymbol{y}(\theta, a) - \boldsymbol{y}(\theta, b) \right) \right] = \boldsymbol{u}^\mathsf{T} \left[\boldsymbol{\beta}_x \Delta f_x + \boldsymbol{\beta}_y \Delta f_y \right] = 0 \tag{9}$$

(10)

Nevertheless, we fit the model of preparatory firing rates given in 8 using regression. We then ask how much of the variance of the observed uniform shift along $\boldsymbol{u}$ can be explained by changes in hand forces, reporting the cross-validated R^2 (Supplementary Table 2).

In this section, we assume that the force axes $\boldsymbol{\beta}_x$ and $\boldsymbol{\beta}_y$ remain the same in the adaptation block. In the next section, we explore whether changes in these force axes themselves could result in the appearance of a uniform shift.

6 Endpoint force tuning

For changes in endpoint force direction tuning, we adapt the model in Eqn. 4 similarly, again allowing each neuron to have different tuning coefficients for the adapted conditions $\alpha_{x,n}$ and $\alpha_{y,n}$.

$$\begin{aligned} y_n(\theta, b) &= \beta_{x,n} f_x(\theta, b) + \beta_{y,n} f_y(\theta, b) + \mu_n \\ y_n(\theta, a) &= \alpha_{x,n} f_x(\theta, a) + \alpha_{y,n} f_y(\theta, a) + \mu_n \end{aligned} \tag{11}$$

Technically, since in this formulation, the norm of the vector $[\alpha_{x,n}, \alpha_{y,n}]$ is allowed to differ from that $[\beta_{x,n}, \beta_{y,n}]$, which allows for the neural tuning gain to change due to adaptation as well as the preferred direction.

If we compute the difference between adapted and before-learning preparatory states, we have:

$$y_n(\theta, a) - y_n(\theta, b) = \alpha_{x,n} f_x(\theta, a) - \beta_{x,n} f_x(\theta, b) + \alpha_{y,n} f_y(\theta, a) - \beta_{y,n} f_y(\theta, b)$$

If we project this difference onto the uniform shift axis, we have:

$$\begin{aligned} \boldsymbol{u}^\mathsf{T} [\boldsymbol{y}(\theta, a) - \boldsymbol{y}(\theta, b)] &= \boldsymbol{u}^\mathsf{T} [\boldsymbol{\alpha}_x f_x(\theta, a) - \boldsymbol{\beta}_x f_x(\theta, b) + \boldsymbol{\alpha}_y f_y(\theta, a) - \boldsymbol{\beta}_y f_y(\theta, b)] \\ &= \boldsymbol{u}^\mathsf{T} \boldsymbol{\alpha}_x f_x(\theta, a) + \boldsymbol{u}^\mathsf{T} \boldsymbol{\alpha}_y f_y(\theta, a) \end{aligned} \quad \boldsymbol{u}^\mathsf{T}\boldsymbol{\beta}_x = \boldsymbol{u}^\mathsf{T}\boldsymbol{\beta}_y = 0$$

If we now marginalize over target direction, we see that changes in preferred directions could potentially drive a consistent shift along $\boldsymbol{u}$ that is common to all conditions, if the new TDR axes $\boldsymbol{\alpha}_x$ and $\boldsymbol{\alpha}_y$ project along $\boldsymbol{u}$ and if there is a non-zero average endpoint force across conditions:

$$\begin{aligned} \mathbb{E}_\theta \left[\boldsymbol{u}^\mathsf{T} [\boldsymbol{y}(\theta, a) - \boldsymbol{y}(\theta, b)] \right] &= \mathbb{E}_\theta \left[\boldsymbol{u}^\mathsf{T} \boldsymbol{\alpha}_x f_x(\theta, a) + \boldsymbol{u}^\mathsf{T} \boldsymbol{\alpha}_y f_y(\theta, a) \right] \\ &= \boldsymbol{u}^\mathsf{T} \boldsymbol{\alpha}_x \mathbb{E}_\theta [f_x(\theta, a)] + \boldsymbol{u}^\mathsf{T} \boldsymbol{\alpha}_y \mathbb{E}_\theta [f_y(\theta, a)] \end{aligned}$$

In our data, changes in force direction axes were small, and therefore unlikely to explain the uniform shift in preparatory states. Nevertheless, we fit the model in Eqn. 11 allowing for altered force tuning using regression. We then again ask how much of the variance of the observed uniform shift can be explained by changes in hand force tuning, reporting the cross-validated R^2 (Supplementary Table 2).

7 Changes in muscle forces (EMG)

Here, we consider the influence of produced muscle forces on the preparatory activity. We use the measured EMG signals for $M = 6$ muscles. To include these as predictors in the regression, we take two approaches. First, we average the peri-movement EMG activation for each muscle, resulting in a per-condition vector of muscle activations $\boldsymbol{m}_{\text{tavg}}(\theta, c) \in \mathbb{R}^M$, where $M = 6$ is the number of recorded muscles. Second, rather than averaging across time, we perform PCA on the $C \times MT$ matrix of EMG activations for the M muscles, C conditions and T time-points, reducing this to $K = 3$ principal components to form a $C \times K$ matrix of muscle response scores. We then use each of the condition-specific rows of this matrix $\boldsymbol{m}_{\text{pca}}(\theta, c) \in \mathbb{R}^K$ as our predictors. In both cases, we have the following functional form for the preparatory activity:

$$\begin{aligned} y_n(\theta, b) &= \sum_{j=1}^{M(\text{or}K)} \beta_{n,j} m_j(\theta, b) + \mu_n \qquad (12) \\ y_n(\theta, a) &= \sum_{j=1}^{M(\text{or}K)} \beta_{n,j} m_j(\theta, a) + \mu_n \end{aligned}$$

where $\boldsymbol{\beta} \in \mathbb{R}^{N \times M}$ or $\mathbb{R}^{N \times K}$ is the matrix of coefficients relating the average muscle activations (or muscle activation scores) to preparatory firing rates for each neuron. If we project the difference in preparatory activity along the uniform shift axis and marginalize over target direction, we have:

$$\begin{aligned} \mathbb{E}_\theta \left[\boldsymbol{u}^\mathsf{T} [\boldsymbol{y}(\theta, a) - \boldsymbol{y}(\theta, b)] \right] &= \mathbb{E}_\theta \left[\boldsymbol{u}^\mathsf{T} \boldsymbol{\beta} [\boldsymbol{m}(\theta, a) - \boldsymbol{m}(\theta, b)] \right] \\ &= \boldsymbol{u}^\mathsf{T} \boldsymbol{\beta} \mathbb{E}_\theta [\boldsymbol{m}(\theta, a) - \boldsymbol{m}(\theta, b)] \\ &= \boldsymbol{u}^\mathsf{T} \boldsymbol{\beta} \Delta \boldsymbol{m} \end{aligned}$$

where $\Delta \boldsymbol{m}$ is the average change in muscle activation (scores) over directions. To assess whether such average changes in muscle activation could drive the uniform shift in preparatory states we observed, we fit a model to preparatory firing rates given by Eqn. 12 using regression. We then ask how much of the variance of the observed uniform shift can be explained by changes in both time-averaged muscle activation and the dimensionality-reduced muscle activation scores, reporting the cross-validated R^2 (Supplementary Table 2).

Lastly, our EMG recordings of muscle activation did not show obvious signs of co-contraction in late-learning, error-clamp and washout trials for the trained target (Extended Data Fig. 4a), consistent with human behavioral studies. Muscle activity in error-clamp trials did not show a uniform shift compared to the before-learning trials, and looked very similar to the before-learning activity for the far untrained targets (Extended Data Fig. 4b).

8 Posture-related changes, gain changes in neural responses

Here we consider whether changes in the animals' posture during or after learning could potentially contribute to the uniform shift. That is, when the body slightly twists, it may change the overall neural activity in the motor cortex (Scott and Kalaska, 1997) and so we might expect these changes to result in a uniform shift or a neural repertoire change throughout the entire trial. Although our animals' postures were not directly measured, we took considerable care to tightly control behavioral variables as discussed in the main text and Supplementary Note 2. To control the overall geometry of the animal's body and arm, we spent many months training them to sit calmly in a nonhuman primate chair (with side walls, neck plate, and a panel through which they could extend their arm to perform the reaching task) that was customized for them. They sat in the same chair at the same height, with the same position of head fixation, and the chair was locked to the haptic device at the same location. Locations of the display screen and the haptic device were also fixed across all days. Though we did not record and quantitatively measure their posture, we did have cameras to monitor their face and body during the experiment. From the cameras we did not see obvious posture changes from the before-learning block to the learning block. This is also consistent with our own observations of each monkey's behavior, which again is part of the standard substantial training process that we have adopted. While it is certainly possible that the monkey could make small changes below the threshold for us to detect, we do not have any reason from observing the animal's behavior to suspect that this could be responsible for explaining the results presented in this manuscript. Moreover, we note that static offsets in position are responsible for a very small fraction of the total variance of neural population activity (Stavisky et al., 2018).

We also note that we did not see a uniform shift during the peri-movement period (Extended Data Fig. 10e, f). Furthermore, we quantified the repertoire change of the baseline neural activity (i.e., when the animal was holding the handle before each trial began at target onset). In contrast to the neural repertoire change during the preparatory period (Fig. 2e, Extended Data Fig. 2e), we did not see a significant neural repertoire change of the pre-trial baseline neural activity (Extended Data Fig. 10b). Because the uniform shift in neural states was observed specifically in preparatory period neural activity, we think that it is unlikely to be driven by postural changes.

Nevertheless, we consider whether posture-related changes in the amplitude or gain of neural responses could drive the uniform shift observed during motor preparation. Gain-related changes in neural firing have been reported accompanying postural changes, i.e., when reaching movements are performed from different initial positions (Caminiti et al., 1991; Pesaran, Nelson, and Andersen, 2006; Batista et al., 2007). These gain related changes have been posited to reflect the dynamic (posture-dependent) relationship between a controlled muscle synergy and spatial direction of the hand (Ajemian, Bullock, and Grossberg, 2001).

A model of preparatory neural firing rates which includes gain modulation that varies from the before-learning state to the adapted state is given by:

$$\begin{aligned} y_n(\theta, b) &= \beta_{x,n} f_x(\theta, b) + \beta_{y,n} f_y(\theta, b) + \mu_n \\ y_n(\theta, a) &= \gamma_n \left[\beta_{x,n} f_x(\theta, a) + \beta_{y,n} f_y(\theta, a)\right] + \mu_n \end{aligned} \tag{13}$$

where γ_n is the adapted condition gain parameter for neuron n.

If we compute the differences in neural activity from the before-learning to adapted conditions, and project this difference onto the uniform-shift axis, we have:

$$\begin{aligned} \boldsymbol{u}^\top [\boldsymbol{y}(\theta, a) - \boldsymbol{y}(\theta, b)] &= \boldsymbol{u}^\top [\boldsymbol{\beta}_x f_x(\theta, b) - \boldsymbol{\gamma} \odot \boldsymbol{\beta}_x f_x(\theta, a) + \boldsymbol{\beta}_y f_y(\theta, b) - \boldsymbol{\gamma} \odot \boldsymbol{\beta}_y f_y(\theta, a)] \quad \Big\rangle\, \boldsymbol{u}^\top \boldsymbol{\beta}_x = \boldsymbol{u}^\top \boldsymbol{\beta}_y = 0 \\ &= \boldsymbol{u}^\top (\boldsymbol{\gamma} \odot \boldsymbol{\beta}_x) f_x(\theta, a) + \boldsymbol{u}^\top (\boldsymbol{\gamma} \odot \boldsymbol{\beta}_y) f_y(\theta, a) \end{aligned}$$

where $\gamma \in \mathbb{R}^N$ is the vector of stacked gain parameters and $\odot$ represents the element-wise Hadamard product. We note that if we define $\boldsymbol{\alpha}_x = \boldsymbol{\gamma} \odot \boldsymbol{\beta}_x$ and $\boldsymbol{\alpha}_x = \boldsymbol{\gamma} \odot \boldsymbol{\beta}_x$, then this is essentially constrained version of the model in Eqn 11, where $\boldsymbol{\alpha}_x$ and $\boldsymbol{\alpha}_y$ are allowed to vary entirely independently of $\boldsymbol{\beta}_x$ and $\boldsymbol{\beta}_y$.

Therefore, neuron-wise changes in gain due to postural changes can stretch the force axes in neural population

axes. If we then marginalize over reach directions:

$$\begin{aligned}\mathbb{E}_\theta\left[\boldsymbol{u}^\top\left[\boldsymbol{y}(\theta,a)-\boldsymbol{y}(\theta,b)\right]\right] &= \mathbb{E}_\theta\left[\boldsymbol{u}^\top\left(\boldsymbol{\gamma}\odot\boldsymbol{\beta}_x\right)f_x(\theta,a)+\boldsymbol{u}^\top\left(\boldsymbol{\gamma}\odot\boldsymbol{\beta}_y\right)f_y(\theta,a)\right]\\ &= \boldsymbol{u}^\top\left(\boldsymbol{\gamma}\odot\boldsymbol{\beta}_x\right)\mathbb{E}_\theta\left[f_x(\theta,a)\right]+\boldsymbol{u}^\top\left(\boldsymbol{\gamma}\odot\boldsymbol{\beta}_y\right)\mathbb{E}_\theta\left[f_y(\theta,a)\right]\\ &= \left(\boldsymbol{u}^\top\boldsymbol{\gamma}\cdot\boldsymbol{u}^\top\boldsymbol{\beta}_x\right)f_x(\theta,a)+\left(\boldsymbol{u}^\top\boldsymbol{\gamma}\cdot\boldsymbol{u}^\top\boldsymbol{\beta}_y\right)f_y(\theta,a)\\ &= 0 \qquad \boldsymbol{u}^\top\boldsymbol{\beta}_x=\boldsymbol{u}^\top\boldsymbol{\beta}_y=0\end{aligned}$$

Consequently, because the force axes are already orthogonalized against the uniform-shift axis, changes in preparatory activity due to gain changes cannot explain the uniform shift we observed. Nevertheless, we fit the model in Eqn. 13 allowing for gain-modulation using regression. We then again ask how much of the variance of the observed uniform shift can be explained by changes in postural gain, reporting the cross-validated R^2 (Supplementary Table 2).

9 Changes in hand speed

To show that the learning-related uniform shift does not relate to the slight change of hand speed after learning (Extended Data Fig. 1d), we expanded the TDR model by adding the hand speed to the regressors (see Methods). Although the hand speed axis explained 10% to 20% of the total variance of the preparatory neural activity (Extended Data Fig. 3b), it was nearly orthogonal to the uniform-shift axis (Extended Data Fig. 3a). The lack of shared variance between the hand speed axis and the uniform-shift axis supports that the uniform shift cannot be simply explained by the change of hand speed.

Supplementary Note 3

Geometry of neural dimensions during the peri-movement period

In the main text, we demonstrated the relationships between neural population dimensions during the preparatory period by computing their pairwise dot products (Extended Data Fig. 3). We found that the TDR 1 + 2 plane largely overlapped with the PC 1 + 2 plane, TDR 1 and 2 axes were nearly orthogonal to the uniform-shift learning axis, and PC 3 was aligned with the uniform-shift learning axis.

In contrast, we did not observe a uniform shift of the peri-movement neural states after learning, but a local shift for reach targets close to the trained target (Extended Data Fig. 10c, d), termed the 'peri-movement local shift'. We took the dot product between the local shift and the TDR 1 and 2 axes during the peri-movement period, and found that this local shift axis significantly overlapped with the TDR axes (Extended Data Fig. 10i). We also found that this shift significantly overlapped with PCs 1-3 (Extended Data Fig. 10i). These results further support the distinction between the peri-movement local shift and the preparatory uniform shift.

Methods

All surgical and animal care procedures were performed in accordance with National Institutes of Health guidelines and were approved by the Stanford University Institutional Animal Care and Use Committee.

The temporal structure of each trial and the curl force field configuration. Two adult male rhesus macaques (*Macaca mulatta*) U (14 kg, 8 years old) and V (10 kg, 8 years old) were trained on the curl force field learning tasks described below. In all tasks, monkeys gripped the handle of a haptic device (delta.3 haptic device, Force Dimension, Switzerland) with their right hands to control the movement of a cursor in a two-dimensional plane displayed on the screen in front of them. The plane within which the monkeys used the haptic device to move their hands was 45 degrees inclined. Monkeys performed a point-to-point delayed reaching task using the haptic device: they initiated each trial by holding their hand within a center target for 450 ms. Then a second target 120 mm (105 mm for monkey U) away from the center showed up on the screen which served as the endpoint of the movement the monkeys were asked to make.

This target originally vibrated in place for a variable delay period (200 – 650 ms, uniformly distributed) and stopped vibrating as a 'go cue' which instructed the monkeys to reach. In curl field trials, the haptic device was programmed to produce forces on the monkey hands as they performed the point-to-point reaching movement. The magnitude and direction of the force depended on the velocity of hand movement according to:

$$\begin{bmatrix} F_x \\ F_y \end{bmatrix} = k \begin{bmatrix} 0 & -1 \\ 1 & 0 \end{bmatrix} \begin{bmatrix} V_x \\ V_y \end{bmatrix} \tag{14}$$

where k was set equal to ±14 N m^{-1} s for monkey V and ±12 N m^{-1}s for monkey U. The sign of k determines the direction of the curl force field: positive k for counterclockwise (CCW) fields and negative k for clockwise (CW) fields. In this study, we applied either a CW or a CCW curl field to the up, down or right reaching target (i.e., six different fields, Extended Data Fig. 1a). In each trial, only one reaching direction had one curl field active. Hand position and velocity were measured at 1k Hz by the haptic device. Hand forces were measured at 30k Hz by a load cell mounted to the haptic device and then down-sampled to 1k Hz during behavior data processing.

Block design of the principal curl force field learning task (single-field learning experiment, Fig. 1a). Throughout the study, a session means a day's worth of experiments, spanning several hours. In every learning session, monkeys first made delayed reaches from the center of the workspace to one of 12 peripheral targets without force field in a before-learning block (block i). Then the learning block (block ii) started where a curl field was active for one reaching target (the trained target). In the learning block, the typical success rate dropped to around 40% at the beginning, and after reaching asymptote, it was around 70% - 80% for monkey U and 80% - 90% for monkey V. After 150 successful learning trials, monkeys showed behavioral adaptation (Fig. 1c) and the task entered the error-clamp block (block iii) where 70% trials were the same as in the learning block and 30% were randomly interleaved error-clamp trials (Albert and Shadmehr, 2018) (20 trials per reaching target).

Error-clamp trials measured whether the newly learned movement kinetics for the trained target were transferred to nearby untrained targets, thereby estimating generalization of learning. The error-clamp block had many more learning trials than the learning block to maintain the adapted behavior. Throughout block iii, to assess feed-forward learning of the curl field, the error clamp was rendered by the haptic device when monkeys reached to each of the same 12 targets as in block i. In these trials, the hand movement was confined to a simulated mechanical channel towards the reach target with a spring constant (stiffness) of 10,000 N/m and damping constant of 150 N m^{-1}s (Howard and Franklin, 2015; Smith, Ghazizadeh, and Shadmehr, 2006). The error-clamp trials were used to probe any change in hand forces for all reach targets without causing visual error feedback because the hand trajectory was ensured to be straight. After the error-clamp block (except for the opposite-field learning experiment, see below) was a washout block (block iv) very similar to the before-learning block in which there was no force field. In all trials throughout all sessions, monkeys were required to finish each reach movement within 700 ms or the trial would be counted as a failure.

In all analyses, early learning was defined as the beginning of the learning block, late learning corresponded to curl field trials in the end of the error-clamp block, after learning corresponded to all error-clamp trials in the error-clamp block, early washout was defined as the beginning of the washout block, and late washout was defined as the end of washout block.

Relearning experiment. To investigate the neural substrate of motor memory retention (the behavioral indicator of which is defined as savings), monkeys were exposed to the same curl field for a second time after washout (monkey U, four sessions; monkey V, three Neuropixels sessions including one M1 recording and two PMd recordings). In these sessions, monkeys did at least 500 (ranging between 539 and 666) washout trials before relearning to make sure that the learned behavior was washed out as completely as possible. The relearning experiment was similar in structure to the single-field learning experiment (see above) except that blocks ii and iii were repeated with the same curl field after the washout block.

Opposite-field learning experiment. To identify and investigate whether the uniform shift was curl-field-specific, in three sessions, monkey U learned two opposite curl fields sequentially for the same reach

direction (up, right or down); in one Neuropixels session, monkey V learned two opposite curl fields sequentially for the same reach direction (up). Monkeys were exposed to each curl field in a learning block and then an error-clamp block. No washout trials were performed between learning the two fields.

Interference experiment design (Fig. 4a, Extended Data Fig. 8a). The interference of learning is defined by the phenomenon that learning one skill (here, one curl field) impedes learning another skill (here, an opposite curl field). The interference experiment was conducted to test the indexing hypothesis that the uniform shift of preparatory neural states may be involved in the separation of motor memories when learning distinct curl fields (Fig. 3) that would otherwise interfere with each other. We expected to see that the preparatory neural states for learning two different curl fields simultaneously would be separated by two distinct uniform shifts, and that the separation of preparatory states would be incomplete if the two fields interfere with each other. In this experiment, monkeys started with a center-out reach block without any curl field (block i). Next in block ii, monkeys experienced either a CCW or a CW field on randomly interleaved trials. The two fields were applied to either the same target, or two targets 30 degrees apart. After around 150 trials of each field, curl-field trials were interleaved with error-clamp trials in the error-clamp block (block iii) and curl-field trials accounted for around 80% of all trials. At last, in the sequential-learning block (block iv), monkeys learned one field for 100 trials and then the other field for another 100 trials. We performed one session of the same-target interference experiment and one session of the two-target interference experiment with each monkey (monkey U and monkey V). Though just two sessions of the interference experiment were performed with each monkey, the results were consistent across monkeys and complimentary to findings when monkeys learned multiple fields sequentially (Fig. 3). Taken together, these results support the indexing hypothesis.

Center-out reach control experiment. This control experiment was conducted to verify that the changes in neural population activity patterns were learning-related, not merely due to the instability of recording. In control sessions (monkey U, n = 3; monkey V, n = 3), monkeys U and V made thousands of delayed center-out reaches to one of 12 targets without any curl force field (70% were no-clamp trials and 30% were error-clamp trials). This control experiment had the same number of blocks as the principal learning task (see above), and each block had approximately the same number of trials to match their corresponding blocks in learning sessions except that the control session did not have a curl field and only had center-out reaches. More concretely, the trial indices for trials in a certain block of the control experiment were within the same range as trials in the same block of the learning experiment (for instance, all error-clamp trials could have trial indices between 500 and 1000 in both learning and control experiments). But the particular trial index of a certain trial type in the learning experiment was not necessarily exactly the same as in the control experiment.

The no-learning control results in Figs. 1d, 2c, 2e and Extended Data Figs. 1c, 2c, 2e, 10e, 10f came from comparing behavioral and neural data in error-clamp trials of block iii and no-clamp trials of block i, to be consistent with trials used in analyses of the learning data. Block ii in control sessions did not have a curl field and so there was no learning.

Random-perturbation control experiment. This control experiment was conducted to verify that the changes of neural population activity patterns were learning-related, not merely because of generating larger muscle forces in a new environment. In one Neuropixels session, monkey V first performed center-out reaches to one of 12 targets without any perturbation force (the before-learning, no-perturbation block). Then in 50% of all center-out reach trials, the haptic device applied a pulse perturbation force, either to the left or to the right of the reaching direction, which simulated the force magnitude of the curl field but the perturbation direction was not predictable (the before-learning, random-perturbation block). Consequently, monkey V could not learn to prepare for the pulse perturbation but instead needed to generate compensatory forces after sensing it. In this block, trials with and without perturbation forces were randomly interleaved. All successful trials in the before-learning no-perturbation block, and perturbation trials in the random-perturbation block were used in data analysis. Last, monkey V experienced the same learning block and error-clamp block as in the single-field learning experiment.

Visuomotor rotation (VMR) experiment. The VMR experiment has been described previously (Vyas, Even-Chen, et al., 2018; Vyas, O'Shea, et al., 2020). Two male adult monkeys, R (15 kg, 12 years old) and J (16 kg, 15 years old), were trained to perform a delayed center-out reach task to one of eight targets. A VMR perturbation was introduced to all eight reach targets during the learning block. Each monkey had two 96-electrode Utah arrays, one implanted in PMd and one in M1. The arrays were implanted five years and seven years for R and J respectively prior to the experiments. In control sessions for the VMR experiment (three sessions per monkey), monkeys R and J made thousands of delayed center-out reaches to one of eight targets without any VMR.

Blinding. Blinding was not relevant for this study because trial parameters within each experiment session were automatically randomly assigned and interleaved without cues for each task block (monkeys could not anticipate the parameters imposed on a given trial), and experimenter did not manually control the parameters used on a given trial. Task blocks were automatically switched once the monkeys achieved a minimal trial count for that block.

Neural data collection (Extended Data Fig. 1b). In a standard sterile surgery, monkey V was implanted with a head restraint and a recording cylinder (19 mm diameter) located over M1 and caudal PMd (stereotaxic coordinates +16 mm anterior, 15 mm lateral) on the left hemisphere. Three Utah arrays (1 mm-long electrodes, spaced 400 m apart, Blackrock Microsystems) were implanted for monkey U eight months prior to the experiments. Extracellular spikes were recorded using three 96-channel Utah arrays implanted in PMd, lateral M1 and medial M1 (monkey U), 24-channel Plexon V-probes (monkey V, version PLX-VP-24-15ED-100-SE-100-25(640)-CT-500), and 384-channel Neuropixels Phase 3A silicon probes (monkey V) (Jun et al., 2017). Central software (v 7.0.2) was used (`https://www.blackrockmicro.com/technical-support/software-downloads/`) for Utah array and V-probe recordings; SpikeGLX software (Imec v 4.3) was used for Neuropixels recordings (`http://billkarsh.github.io/SpikeGLX/`). In this study, monkey U results included 12 recording sessions (four sessions of the relearning experiment, three sessions of the opposite-field experiment, two sessions of the interference experiment, and three sessions of the center-out control experiment); monkey V results included 10 Neuropixels recording sessions (three sessions of the relearning experiment, one session of the opposite-field experiment, two sessions of the interference experiment, three sessions of the center-out control experiment, and one session of the random-perturbation experiment) and two sets of V-probe recordings for learning two different curl fields (20 sessions in total). Out of the 13 learning datasets (monkeys U and V), three had curl fields applied to a right reaching target and 10 had curl fields applied to an up or down target. Although more datasets had a curl field applied to the up or down target than to the right target, we believe that this did not bias the results in a target-dependent fashion because we observed consistent results across sessions.

Neural data pre-processing. For Utah array recordings, voltage signals were band-pass filtered from each electrode (250 Hz – 7.5 KHz). These signals were processed to detect 'threshold crossing' spikes. We detected spikes whenever the voltage crossed below a threshold of -3.5 times the root-mean-square voltage. For V-probe and Utah-array recordings, spike sorting was performed offline using a custom software package (Kaufman et al., 2014) (available online as MKsort; `https://github.com/ripple-neuro/mksort/`); stable single units and multi-unit isolations were included. Around 400 units from 20 V-probe recording sessions (22 ± 9 units per session) were sorted by MKsort and used in the analysis. Around 245 ± 34 single- and multi-units from the three Utah arrays passed the sorting criteria in each session. For Neuropixels recordings, the original data were automatically spike sorted with the Kilosort spike sorting software and then manually curated with the phy tool (`https://github.com/kwikteam/phy`). More than 1000 units from 10 Neuropixels recording sessions (149 ± 20 units per session) were sorted by KiloSort and 'phy' and used in the analysis. For V-probe recordings, neurons recorded over multiple sessions were pooled together for the same curl field applied to the same reach direction because these sessions shared a common task structure and configuration, which resulted in at least 100 units per curl field. Despite the various spike-detection or sorting methods, we acquired consistent analysis results.

The V-probe and Utah array recordings included both single and multi-unit isolations, and after spike sorting, we kept stable single units and multi-units. Approximately 40% of all sorted Utah-array units were single

units while for V-probe recordings the percentage was around 50%. Neuropixels probes could record single units with the currently highest quality to our knowledge. Among all recorded units, the portion of stable single units was usually between 40% and 70%. After spike sorting, at least 80% of all sorted Neuropixels units were single units.

EMG data collection and pre-processing. For monkey U, surface EMG recordings (Trigno EMG Systems, Delsys Inc.) were made from triceps brachii, biceps brachii and posterior deltoid. For monkey V, surface EMG recordings were made from the trapezius, lateral deltoid, pectoralis, biceps brachii, extensor carpi radialis and flexor carpi radialis. EMG signal was processed by the signal envelopes taking the upper and lower peaks smoothed over 80-sample intervals.

Data analysis. The behavioral, EMG, and neural data were analyzed offline using MATLAB 2017b and 2019a (MathWorks). All analyses pooled together PMd and M1 recordings and used the full-dimensional neural data, unless otherwise specified in the following sections or figure legends.

Behavior quantification. To examine behavioral learning, generalization, and washout, two measures were used: (1) kinematic error (the lateral hand trajectory deviation error), and (2) the kinetic change (hand force difference). The kinematic error was calculated on each reaching movement as the perpendicular error of the hand path relative to a straight line joining the center and the target locations of the movement, averaged over a 20 ms window around the peak speed. For both monkeys, the kinematic error in all blocks of each session was a rolling average over 10 trials to reduce the single-trial noise while preserving the temporal resolution of learning and washout, with the sign flipped appropriately so that errors in CW and CCW field trials could be combined. The mean and s.e.m. of the kinematic error were then computed across all learning sessions (Fig. 1c). The kinetic change was calculated as the perpendicular hand force difference (the compensatory force) between error-clamp trials and their corresponding before-learning trials for the same reach target (averaged over 100 - 200 ms from movement onset). Note that throughout the study, perpendicular indicated perpendicular to the reach direction.

Principal component analysis (PCA) and time window selection. To explore neural population activity patterns, we used PCA, an unsupervised dimensionality reduction method that operates on neural data alone and extracts neural dimensions explaining the largest amount of variance. To perform PCA on neural data, we constructed the data matrix R (Conditions $\times$ Time) $\times$ Neurons, where conditions are defined by reaching directions and force field type. Time points were spaced 1 ms apart for neural trajectory analyses or averaged over a 100 ms bin within the chosen time window for neural state analyses. Preparatory period was the time window -50 to +50 ms from the go cue for all neural state analyses and -500 to 200 ms for neural repertoire analyses. For peri-movement we used the time window 0 to +100 ms from movement onset for all neural state analyses and 0 - 600ms for neural repertoire analyses. The neural trajectory of each trial was defined by -150 to +150 ms from target onset, -50 to +50 ms from the go cue, and -200 to +400 ms from movement onset. Movement onset for each reach was determined via a speed threshold of 35 mm/s for monkey V and 40 mm/s for monkey U. Spike counts were lightly smoothed and averaged across all trials for each condition, and the activity of a given neuron was centered by subtracting its mean response from the firing rate at each time point in each condition. The condition-averaged neural data matrix was passed to the SVD function to compute the principal components (PCs). The dimension that captures the greatest amount of neural variance is principal component dimension 1 (PC 1), the second most is component 2 (PC 2), and so forth. Importantly, the PCs are orthogonal to each other.

For the neural state analysis, we chose time windows -50 to +50 ms from the go cue and 0 to +100 ms from movement onset, because the former was right before peri-movement activity rose (i.e., near the end of the preparatory period, with stronger neural tuning than the early stage of the preparatory period) and the latter was in the early stage of the peri-movement period before sensory feedback signal came to the motor cortex (i.e., mainly motor signal). For the neural repertoire analysis, we chose the time windows -500 to -200 ms and 0 to 600 ms from movement onset and averaged the neural activity every 100 ms, because we wanted to denoise the data (by averaging over time) and meanwhile generate multiple before- and after-learning states for each reach direction (by choosing a larger time window) to more thoroughly represent the before-

and after-learning neural pattern repertoires. The neural trajectories in PCs 1-4 (Extended Data Fig. 10a) showed the following patterns in the time widows we chose:

1. The -50 to +50 ms time window around the go cue, indicated as the gray area, was within the preparatory period and had strong neural tuning reflected by the first three PCs.
2. The 0 to +600 ms and 0 to +100 ms time windows from movement onset chosen for peri-movement neural repertoire and neural state analyses had strong peri-movement activity.
3. Peri-movement activity started to rise around 150 ms before movement onset. Thus, the -500 to -200 ms time window from movement onset for the preparatory neural repertoire analysis was within the preparatory period.

Targeted dimensionality reduction (TDR). We used TDR (Mante et al., 2013) to identify low-dimensional subspaces capturing variance related to the behavioral variables of interest. To construct the TDR space, we used multivariable linear regression to determine how various behavioral variables affected the responses of each unit. The neural data were first averaged across all trials for each condition, and the activity of a given neuron was centered by subtracting its mean response from the firing rate at each time point in each condition. We then described the centered responses of neuron i as a linear combination of several behavioral variables:

$$r_i(k) = \beta_{i,0} + \beta_{i,x} F_x(k) + \beta_{i,y} F_y(k) + \sum_{j=1}^{n} \beta_{i,j} \mathrm{Ind}_j(k) \tag{15}$$

where $r_i(k)$ is the centered trial-averaged response of unit i on condition k, averaged over a certain time window (same time windows as in the PCA procedure). $F_x(k)$ and $F_y(k)$ are the trial-averaged horizontal and vertical hand forces on condition k. $\mathrm{Ind}_j(k)$ is a binary indicator of the trial type: it is 1 if condition k is the error clamp trial for curl field j and 0 otherwise. The regression coefficient $\beta_{i,0}$ captures variance independent of the listed behavioral and task variables. The 2D force-predictive subspace was built by regressing full-dimensional preparatory neural activity against initial hand forces without binary indicators (i.e., $n = 0$), using only before-learning trials. Initial hand forces were defined as hand forces averaged over the first 50 ms following movement initiation, which reflected the feed-forward control of the prepared movement before sensory feedback arrived to motor cortex (Hatsopoulos and Suminski, 2011). To study the hand speed-related neural dimension, we expanded this 2D TDR model with the hand speed as an additional behavioral variable, using before-learning and error-clamp trials. This expanded model had an additional regressor $\beta_{i,\mathrm{speed}} v(k)$ added to the right side of the equation, with $n = 0$ and $v(k)$ being the maximal hand speed on condition k:

$$r_i(k) = \beta_{i,0} + \beta_{i,x} F_x(k) + \beta_{i,y} F_y(k) + \beta_{i,\mathrm{speed}} v(k) \tag{16}$$

Using equation 15, we also built a 3D TDR model that incorporated an indicator of the trial condition (before-learning trials vs. after-learning error-clamp trials) as an additional regressor (i.e., $n = 1$), using before-learning and error-clamp trials. This model revealed a uniform shift during learning along the third dimension similar to the PCA results (Extended Data Fig. 2h, 2i).

To estimate the regression coefficients, we constructed the following matrix M with shape conditions $\times$ regressors. All but the first column of M contained the condition-by-condition values of one of the behavioral / task variables (the regressors). The first column consisted only of ones to estimate β_0. Given the conditions $\times$ neurons matrix of neural firing rates R, the regression model could be written as:

$$R = M \begin{bmatrix} \beta_0 & \beta_x & \beta_y & \beta_1 & \beta_2 & \dots & \beta_n \end{bmatrix}^\intercal \tag{17}$$

Then the regression coefficients could be estimated as:

$$\begin{bmatrix} \beta_0 & \beta_x & \beta_y & \beta_1 & \beta_2 & \dots & \beta_n \end{bmatrix}^\intercal = (M^\intercal M)^{-1} M^\intercal R \tag{18}$$

We then projected neural data into the regression subspace by multiplying the pseudoinverse of the coefficient matrix with the neural data matrix R.

Measurement of changes to the neural repertoire. We applied the approach described previously (Golub et al., 2018) to the top 10 PCs of the neural population data. Briefly, we quantified the neural repertoire change as the distances between each after-learning neural activity state in the error-clamp block and its nearest neighbors among all the before-learning states, normalized by the variance of the before-learning neural state repertoire. Values near zero indicated repertoire preservation and larger values indicated repertoire change. We measured and compared the neural repertoire changes for curl field learning sessions (using before-learning and error-clamp trials), center-out control experiment sessions (using center-out reach trials in block i and block iii of the center-out control experiment), and random-perturbation experiment sessions (using before-learning no-perturbation trials, before-learning random-perturbation trials, and after-learning error-clamp trials).

Definition of uniform-shift axes and quantification of neural-state uniform shift. In the visualization of preparatory neural states projected to the first three PCs (Fig. 2d), we observed that all the after-learning states were separated from the before-learning states (a uniform shift). To determine a neural axis that best captures this uniform shift, as this need not align precisely with PC 3, we defined uniform-shift axes by the following steps. We took the centroids of preparatory states in the full-dimensional neural space of all reach directions in the before-learning block, after-learning error-clamp block and late-washout block. The axis that connected before-learning and after-learning centroids was defined as the 'uniform-shift learning axis'; the axis that connected after-learning and washout centroids was defined as the 'uniform-shift washout axis'.

We orthogonalized each uniform-shift axis against the force-predictive TDR axes where we found rotatory neural shifts, before we did any quantification analysis of the uniform shift. Orthogonalization was a necessary process allowing us to examine a neural dimension that did not involve the TDR axes which we already investigated. The uniform-shift axes were also always orthogonalized relative to each other before we projected and plotted preparatory neural states on them (see Figs. 3a-c, 5e and 5i). The statistical test of Fig. 2d was performed on the before- and after-learning neural states of all reach directions projected onto the uniform-shift learning axis. In Fig. 5d, 5j and Extended Data Figs. 2d, 9b, we projected full-dimensional neural population activity in learning, washout and relearning trials of the trained target onto the uniform-shift learning axis to quantify their distance from the before-learning state along this axis. Fig. 5i showed centroids of the late-washout and late-relearning neural states projected onto the uniform-shift learning and washout axes (seven sessions, monkeys U and V). Normalization was performed against the distance between before- and after-learning neural state centroids in each session (see Fig. 5d, 5i, 5j, and Extended Data Figs. 2d, 9b).

Measurement of geometric relationships between uniform-shift axes. We took the dot products between different uniform-shift axes as defined above to measure their geometric relationships: values close to 1 indicated that the two axes were close to parallel, -1 indicated an antiparallel relationship, and 0 indicated orthogonality. To quantitatively test for these geometric relationships, we compared the observed uniform shifts to control distributions generated for each relationship. Each control distribution was defined by a set of before- and after-learning preparatory states resampled from the observed data, and computed in the following ways to reflect the corresponding geometric relationship:

- *Parallel / antiparallel case:* for each learned curl field, we resampled its before-learning and after-learning trials and measured the dot products between all the resampled uniform-shift axes. Without noise intrinsic to the data, these resampled uniform-shift axes for the same curl field should be truly parallel and the dot product should be 1. The antiparallel distributions were constructed from taking the inverse of the parallel distributions but with a separate independent set of resampling.
- *Orthogonal two-field case:* we first used all trials of learning two different curl fields applied to two different reach targets to define their trial-averaged uniform-shift axes, as described in the last section. We orthogonalized these two trial-averaged uniform-shift axes, denoted as $\bar{v}_1$ and $\bar{v}_2$. We then resampled trials of learning these two fields: in the ith round of resampling, we defined and orthogonalized the resampled uniform-shift axes for the two fields, denoted as $\tilde{v}_{1,i}$ and $\tilde{v}_{2,i}$, $i = 1, 2, \ldots, n$. We measured the dot products between $\bar{v}_1$ and $\tilde{v}_{2,i}$, as well as between $\bar{v}_2$ and $\tilde{v}_{1,i}$, because without the resampled estimation noise, they should be truly orthogonal and the dot product should be 0.

- *Orthogonal washout case:* we applied the same procedure as in the orthogonal two-field case, except that we used learning and washout trials of one single field rather than learning trials of two different fields.

Measurement of relationships between neural population dimensions (Extended Data Fig. 3a). Relationships between different neural population dimensions - the TDR subspace, the uniform-shift axis and the PC subspace - were measured by taking the pairwise dot products between their projection vectors (i.e., the vectors that project the full-dimensional neural data onto the lower-dimensional neural subspaces). For instance, the 2D TDR subspace was associated with two projection vectors estimated from the linear regression, the uniform-shift axis itself was a projection vector, and the PC subspace had eigenvectors as the projection vectors. A dot product close to 1 indicates that two dimensions are closely aligned whereas a dot product close to 0 indicates that two dimensions are nearly orthogonal.

Minimum distance decoder. The minimum distance decoder used half of all trials as training trials to find the centroids of before-learning, after-learning and washout preparatory neural states, and decoded the condition type (before-learning, after-learning or washout for a given curl field) and the curl field type based on to which centroid the neural state vector of the test trial was closest (i.e., smallest Euclidean distance). The decoding performance was evaluated by cross validation.

Interference experiment data analyses (Fig. 4 and Extended Data Figs. 7, 8). The TDR analysis of the interference data was conducted in the same way as described previously in the Targeted dimensionality reduction (TDR) section. We used trial-averaged neural and behavioral data from block i before-learning trials and block iii error-clamp trials to build the model. We projected neural activity in all blocks into the force-predictive TDR subspace to visualize their patterns (Fig. 4d, Extended Data Figs. 7c, 8c).

We defined the two field-specific uniform shifts as the vector connecting the centroid of all error-clamp neural states to the late sequential-learning state (the average of last 20 trials) of the first field, and the vector connecting the late sequential-learning state of the first field to the late sequential-learning state of the second field. We then orthogonalized both uniform shifts against the TDR axes. To visualize these two uniform shifts, we orthonormalized them and projected preparatory neural activity onto these two orthogonal axes (Fig. 4e, Extended Data Figs. 7d, 8e).

We found a residual shift during interference as the vector connecting the centroid of before-learning states to the centroid of error-clamp states, which was orthogonalized against the TDR axes and the two field-specific uniform shifts. We leave it for future work to systematically address the functional roles of the residual neural shift during interference.

Measurement of neural trajectory distance (Fig. 5h, Extended Data Fig. 9d, 9h). We first averaged the neural trajectories across trials for each condition. To estimate the neural trajectory distance between different conditions over time, we performed a Euclidean distance analysis as follows: for the two neural trajectories we were comparing, we quantified the Euclidean distance between pairs of points during the preparatory period (-50 to +50 ms from the go cue) and during the movement period (+100 to +200 ms from movement onset), in the first three PCs. The first three PCs accounted for around 60% - 70% of the variance of the neural trajectory data in all data sets. A smaller distance indicated that the two trajectories were more similar.

Measurement of relative neural trajectory similarity (Extended Data Fig. 9h). The relative neural trajectory similarity was a metric to compare whether the washout neural trajectory was more similar to the before-learning or the after-learning trajectory. It was quantified as the neural trajectory distance (see the last section) between washout and after-learning trajectories over the distance between washout and before-learning trajectories during a certain time window. A larger value indicated higher similarity to the before-learning neural trajectory than to the after-learning neural trajectory. Control analyses measured this metric by splitting all before-learning trials into two random halves, with one half treated as the before-learning trials and the other serving as the sham washout trials. We combined data from two monkeys (seven

relearning-experiment sessions from monkeys U and V) to gain a higher statistical power for the one-sided Wilcoxon rank-sum test.

Tracking neurons over multiple sessions. To examine the relationship between uniform shifts of learning multiple force fields, we selected neurons that showed up in the same Utah array channels over five successive days. We evaluated the cross-day similarity of waveforms for each sorted neuron by (1) binning the waveform data into a 40-D vector for each neuron per session, and (2) calculating the Pearson correlation coefficient between waveforms on day 1 vs. day 2, day 1 vs. day 3, and so on (Fraser and Schwartz, 2012). The null correlation coefficient was generated from comparisons between waveforms that were known to be from different neurons (i.e., neurons from separate channels). Neurons with a significantly higher cross-day waveform correlation than the null correlation were selected (i.e., higher than 95% quantile of the null correlation). At last, we confirmed the stability of the selected neurons by visualizing their waveforms over days. Cross-day waveforms and PSTHs of example neurons were shown in Extended Data Fig. 6a, b.

Minimum trial count for learning or relearning (Fig. 5j, k). We first calculated the compensatory hand force (hand force perpendicular to the reach direction) within 100 ms after movement onset in each trial, because learning was marked by an earlier increase of the compensatory force (Extended Data Fig. 1d). The 'minimum trial count for learning or relearning' was then measured as the trial count when this compensatory force during learning or relearning first achieved 90% of the same force averaged over the last 50 successful learning trials.

Definitions of behavioral terminology

Based on Krakauer et al. (2019).

Motor learning. Motor learning encompasses a wide range of behavioral phenomena, from behavioral calibration (such as adaptation), to de novo learning of new skills and to making high-level cognitive decisions about how to act in a novel situation. Consistent with this definition, we think the process of learning new arm forces in the curl field is one type of motor learning, in particular the adaptation of muscle dynamics to the new force environment (Extended Data Figs. 1d, 4a).

Skill acquisition vs. skill maintenance. We adopt a two-part operational definition of motor learning: (1) skill acquisition is the process by which an individual acquires the ability to rapidly identify an appropriate movement goal given a particular task context, select the correct action given a sensory stimulus and / or the current state of the body and the world, and execute that action with accuracy and precision; (2) skill maintenance is the ability to maintain performance levels of existing skills under changing conditions. These two aspects of motor learning are each important in their own right, and they likely share overlapping neural circuitry. That said, it is also clear that the brain possesses dedicated mechanisms for skill maintenance.

Adaptation. Adaptation is the adjustment of behaviors to a new task condition or environment, which involves task switching (here, switching to the new force environment). Adaptation is considered to be one type of learning aimed at skill maintenance when one encounters changes in the body or environment. It is usually compared to de novo learning, i.e., the acquisition of completely new skills from scratch. In our experiment, the animals on average experienced the new force environment for around 1 hour every day and they showed strong aftereffect in the washout block, which demonstrated the process of adaptation.

Definitions of neural terminology

Neural population state. The N-dimensional coordinates (state) of a population of N neurons found by counting the number of action potentials emitted from each neuron within a time bin. The neural population state is then an N-dimensional point, or a lower dimensional point if a dimensionality reduction method is applied to the data. Finally, if multiple trials are averaged together, the average neural population state retains the same definition except that the average number of action potentials emitted from each neuron within a time bin is used.

Initial condition. The preparatory neural population state at the time of the 'go cue' in an instructed-delayed reach task.

Changes in neural population state. The difference between the neural population states before and after some perturbation, in this case the application of a curl field. In the curl field learning experiments the changes in neural population state can be (1) during learning (i.e., how the preparatory neural population state changes from trial to trial during the application of a curl field) or (2) after complete learning (i.e., after numerous curl field trials such that the after-learning preparatory neural state becomes steady). Changes in initial condition is a specific case of changes in neural population state at the time of the go cue during the preparatory period.

Neural population dynamics. This typically refers to the evolution of the neural population state over time within a trial (i.e., a fairly fast timescale).

Changes in neural population dynamics. This refers to changes in how the neural population state evolves. For example, if we use the standard equation $\frac{dx(t)}{dt} = Ax(t) + Bu(t)$ to describe neural population dynamics, changes in neural population dynamics can be represented as the change of matrices A or B across trials (i.e., across longer timescales), where A and B are assumed to be fixed when studying fast timescale neural population dynamics, but in reality A and B are functions of time that come into play across longer timescales and are especially likely to change during learning. While the timescale of neural population dynamics itself is typically as short as within a single trial, changes in neural population dynamics typically happen over longer timescales, across multiple trials and minutes or longer. We use 'changes in neural population state' and 'changes in neural population dynamics' to mean longer-timescale neural changes across trials (as accompany learning), and we use 'neural population dynamics' as we have in prior publications when talking about the evolution of neural state over time within a trial.

New neural activity patterns. Our finding of the neural repertoire change suggests the formation of new neural population activity patterns after learning the curl field. What we mean by "new" is that those neural patterns are specific to moving in a curl field and are not used for a standard set of behaviors such as the center-out reaches or correcting random perturbations. It seems possible that the capacity to exhibit those neural patterns may have already been present prior to learning and the animals did not express them in a simpler reaching task. But in our definition, those patterns are still new compared to the neural patterns used in the standard reaching movements before learning the curl field.

Re-aiming and reassociation. Reassociation (Golub et al., 2018) is the neural strategy of learning subject to a fixed, existing repertoire of neural activity patterns. In other words, the neural activity patterns used for a particular behavior change during learning, but remain within the set of neural activity patterns that could be generated by (or empirically observed from) the neural population before learning. Here, the control space is the neural repertoire. Re-aiming is the neural strategy of learning whereby the neural activity patterns used for a given behavior after learning are the same neural activity patterns used for a potentially different behavior before learning. Here, the control space is the behavioral repertoire. Because the neural activity patterns used after learning are part of the existing neural repertoire, re-aiming is a subset of reassociation. Re-aiming has been observed during learning in VMR tasks (Vyas, Even-Chen, et al., 2018; Vyas, O'Shea, et al., 2020; Jarosiewicz et al., 2008; Chase, Kass, and Schwartz, 2012), whereby the perturbation applied to the effector is countered by a rotatory shift in neural activity patterns. The rotatory shift of preparatory states we observed in the 2D TDR subspace resembled these re-aiming findings. However, we referred to these types of changes as reassociation-like (as opposed to re-aiming-like) because (1) preparing for a compensatory force to oppose the curl field could result in re-aiming-like rotatory shifts even if the motor system does not explicitly re-aim for a nearby reaching target, (2) curl field learning likely demands more than purely re-aiming which is sufficient in VMR learning, (3) we can directly test for reassociation (based on repertoire change, as in Golub et al. (2018)), and (4) all re-aiming-like changes are consistent with reassociation.

Statistics

To test the correlation between TDR-predicted hand forces and real hand forces during learning, we fitted regression curves between them (Fig. 2a, Extended Data Fig. 2a). To test the single-trial learning and washout of neural states, we fitted regression curves of neural shifts against the trial count (Fig. 5c, 5d; Extended Data Figs. 2d, 9a, 9b). To test the trend of gradual learning and gradual washout of neural states binned over trials, we applied the Cuzick's test (Extended Data Fig. 10d, h). It is Cuzick's extension of the one-sided Wilcoxon rank-sum test to assess trend in data with three or more ordinal groups. Because we did not assume that the data followed a normal distribution, we applied the Wilcoxon rank-sum test to compare groups of data and signed rank test to compare a group with a null mean value, using the one-sided test where appropriate. We repetitively subsampled the original data to generate before- and after-learning neural states to acquire control distributions of the dot products of uniform-shift axes (see above for the generation of control distributions). We used Hotelling's T2 test, a multivariate probability distribution tightly related to the F-distribution, to compare high-dimensional neural states between different blocks (e.g., Fig. 5e, Extended Data Fig. 5a, b).

For all tests, we used $P = 0.05$ as the significance threshold, with * for $P < 0.05$, ** for $P < 0.01$, and *** for $P < 0.001$. Error bars were due to quantification from multiple trials per condition, from multiple sessions, or from multiple reach directions × multiple sessions.

Total number of curl-field sessions. In this study, monkey V had a month of behavioral training with a 10 N/(m*s^{-1}) curl field without neural recording. We then did 40 sessions of V-probe recordings in total, of which 20 sessions were used in the analyses because in those 20 sessions monkey V completed sufficient trials in all blocks to enable our analyses. Out of the 12 Neuropixels sessions with curl fields, our analyses used three sessions of the single-field learning / relearning experiment, one session of the random-perturbation experiment, one session of the opposite-field learning experiment, and two sessions of the interference experiment. Not all sessions with curl field learning were analyzed, and we excluded the following for monkey V: behavioral training sessions (no neural recordings), V-probe sessions without enough trials finished, and Neuropixels sessions without enough active stable units or enough trials finished.

With monkey U, we conducted seven sessions of behavioral training with a 10 N / (m*s^{-1}) curl field before we started neural recording. We then did 12 sessions of Utah-array recordings (three implanted arrays, each with 96 electrodes) with curl fields, among which we analyzed four sessions of the single-field learning / relearning experiment, three sessions of the opposite-field learning experiment, and two sessions of the interference experiment. Not all sessions with curl field learning were analyzed, and we excluded the following for monkey U: behavioral training sessions (no neural recordings); recording sessions without enough trials finished.

Monkey V showed significantly faster learning in late neural recording sessions than in early behavioral training sessions (not shown). Despite this learning rate difference, other behavioral variables we measured and the neural patterns we investigated in monkey V looked largely the same in earlier V-probe and later Neuropixels recording sessions. Monkey V results in this manuscript were all from the late neural recording sessions. The behavior of monkey U looked largely the same in behavioral training sessions and neural recording sessions. The neural patterns we investigated in monkey U also looked largely the same in earlier and later neural recording sessions.

Datasets and sessions in each figure. *Grouped data:* in Fig. 5i, 5j (right panel) and Extended Data Figs. 5e, 9h, 10i, we grouped together monkey U and monkey V sessions rather than analyze them separately to gain higher statistical power for performing the signed-rank test. Example EMG recordings from both monkeys were grouped together (Extended Data Fig. 4). Note that each panel of the EMG figure was from one specific muscle of one monkey, but the 6 total recorded muscles were either from monkey U or monkey V.

Data from only one monkey: The random perturbation experiment was performed only with monkey V (Fig. 2f, 2g and Extended Data Fig. 5c). We were able to track the same neurons over five sessions only in monkey U (using three implanted Utah arrays) to study the uniform shifts during multi-session multi-field learning (Fig. 3b, 3c, 3d field 1 vs. 2, Extended Data Fig. 6c distinct fields), the relationship between the uniform

shift and learning rate (Fig. 5k), and the uniform shifts for the same field learned in two sessions (Extended Data Fig. 9f). The stability of representative single units across these sessions was shown in Extended Data Fig. 6a, 6b.

Data from both monkeys shown separately: all the other results. Note that plots of example single-trial hand trajectories, visualized neural states and visualized neural trajectories were from an example session in one monkey and consistent across sessions in both monkeys; the behavioral and neural quantification results were shown separately for both monkeys unless otherwise specified. The interference experiment results (Fig. 4 and Extended Data Figs. 7, 8) came from two sessions with each monkey. The VMR experiments were collected in two monkeys and analyzed separately as noted in the "Visuomotor rotation (VMR) experiment" section of Methods (Extended Data Fig. 2f, g).

Supplementary Discussion

In this study, we discovered uniform shifts of preparatory activity along learning-related orthogonal dimensions that appeared to index motor memories in neural state space. In a large neural population, many orthogonal dimensions are available in neural space; however, neural activity during movement is typically thought to occupy a lower dimensional manifold within that space. Our results here demonstrate that motor cortex engages specific, readily available orthogonal dimensions to index motor memories, adding to a body of prior findings that demonstrate neural dimensions to organize internal computations and facilitate pattern generation (Kaufman et al., 2014; Hennequin, Vogels, and Gerstner, 2014; Sussillo et al., 2015). This highlights new avenues to better comprehend computation through neural population dynamics, and underscores the need to dissect the geometric organization of multiple behaviors, task-related computations, and contextualized motor memories in a shared neural state space (Duncker, Driscoll, Shenoy, et al., 2020).

Previous work showed that the preparatory neural activity mainly occupies the 'output-null' subspace of neural population activity in PMd and M1 (Kaufman et al., 2014), but it remained unknown if all output-null activity is tightly coupled to movement output parameters. In this work, the uniform shift of preparatory neural states occurred to reach directions where the movement was unaltered during learning, which indicates that uniform shifts and the resulting new activity patterns not only live in the output-null subspace (Kaufman et al., 2014), but also reflect components of the output-null activity that do not directly relate to the movement output. Perich and colleagues recently reported neural changes in the 'M1-null' subspace of PMd for rapid learning, where the M1-null subspace represents the component of the PMd population activity that does not directly influence M1 population activity (Perich, Gallego, and Miller, 2018). Is the uniform shift in our work confined to the M1-null subspace of PMd? By investigating PMd and M1 neural populations separately, we identified a highly similar reassociation subspace and the uniform shift axis as when we analyzed both areas jointly (not shown). Our results suggest that the uniform shift is not confined to only PMd, but it is confined to the preparatory neural activity in both PMd and M1 and does not occur during the peri-movement period (Extended Data Fig. 10c-i). Perhaps the uniform shift we found here is a different type of learning-related change: it lives in the 'muscle-null' subspaces of PMd and M1 and may not necessarily be confined to the M1-null subspace of PMd. Alternatively, it is possible that most M1 neurons in Perich, Gallego, and Miller (2018) did not have significant preparatory activity and consequently the preparatory neural changes they found were only confined to PMd. While there is no inconsistency between our findings and those by Perich, Gallego, and Miller (2018), further work is needed to precisely relate these studies.

Furthermore, the occurrence of uniform shifts provides evidence for the formation of new activity patterns during short-term motor learning that are not used in standard reaching movements. Previously, the circuit structure or connectivity of an existing network has been thought to constrain the patterns that its neurons are capable of exhibiting, which may limit its capacity for short-term learning (Sadtler et al., 2014; Oby and Yu, 2017; Sakellaridi et al., 2019). Here our results suggest that the motor system may be more flexible than previously thought, and can generate new activity patterns specific to the new movements not only during long-term learning (Oby, Golub, et al., 2019) but also within one day in order to quickly adapt to a changing environment. To adapt to the new force environment when learning a curl force field, subjects need to acquire new movement kinetics (Extended Data Fig. 1d). This task demand is a major

feature differentiating curl field learning from other short-term learning contexts in which reassociation of existing neural activity patterns is sufficient to support behavioral learning without neural repertoire changes (Jarosiewicz et al., 2008; Golub et al., 2018; Vyas, Even-Chen, et al., 2018; Chase, Kass, and Schwartz, 2012; Sakellaridi et al., 2019; Hwang, Bailey, and Andersen, 2013). The observation of reassociation in different motor learning contexts suggests that motor cortical neurons may try reassociating existing neural patterns first when learning new behaviors, and if reassociation alone is not sufficient to complete learning, the motor system may engage different learning processes and new neural activity patterns.

In future work, it may be possible to directly and specifically perturb the uniform shift by precisely manipulating the neural activity (i.e., modulate specific neural population dimensions), which could provide a causal test of the indexing hypothesis (Marshel et al., 2019; Chettih and Harvey, 2019; Carrillo-Reid et al., 2019; Robinson et al., 2020). Motor learning engages a brain-wide network spanning other cortical regions, thalamus, cerebellum, basal ganglia, brainstem, and the spinal cord (Krakauer et al., 2019; Bastian, 2006; Herzfeld et al., 2014; Albouy et al., 2008). The highly structured population geometry accompanying learning in the motor cortex likely reflects the collective action of these distributed regions, and opens many questions about their functional interactions that facilitate motor learning.

References

Afshar, Afsheen et al. (Aug. 2011). "Single-trial neural correlates of arm movement preparation". In: *Neuron* 71.3, pp. 555–564. ISSN: 0896-6273, 1097-4199. DOI: 10.1016/j.neuron.2011.05.047 (cit. on p. 2).

Ajemian, R, D Bullock, and S Grossberg (Dec. 2001). "A model of movement coordinates in the motor cortex: posture-dependent changes in the gain and direction of single cell tuning curves". en. In: *Cerebral cortex* 11.12, pp. 1124–1135. ISSN: 1047-3211. DOI: 10.1093/cercor/11.12.1124 (cit. on p. 9).

Albert, Scott T and Reza Shadmehr (Apr. 2018). "Estimating properties of the fast and slow adaptive processes during sensorimotor adaptation". en. In: *Journal of neurophysiology* 119.4, pp. 1367–1393. ISSN: 0022-3077, 1522-1598. DOI: 10.1152/jn.00197.2017 (cit. on p. 11).

Albouy, Geneviève et al. (Apr. 2008). "Both the hippocampus and striatum are involved in consolidation of motor sequence memory". en. In: *Neuron* 58.2, pp. 261–272. ISSN: 0896-6273, 1097-4199. DOI: 10.1016/j.neuron.2008.02.008 (cit. on p. 22).

Bastian, Amy J (Dec. 2006). "Learning to predict the future: the cerebellum adapts feedforward movement control". en. In: *Current opinion in neurobiology* 16.6, pp. 645–649. ISSN: 0959-4388. DOI: 10.1016/j.conb.2006.08.016 (cit. on p. 22).

Batista, Aaron P et al. (Aug. 2007). "Reference frames for reach planning in macaque dorsal premotor cortex". en. In: *Journal of neurophysiology* 98.2, pp. 966–983. ISSN: 0022-3077. DOI: 10.1152/jn.00421.2006 (cit. on p. 9).

Caminiti, R et al. (May 1991). "Making arm movements within different parts of space: the premotor and motor cortical representation of a coordinate system for reaching to visual targets". In: *The Journal of neuroscience: the official journal of the Society for Neuroscience* 11.5, pp. 1182–1197. ISSN: 0270-6474 (cit. on p. 9).

Carrillo-Reid, Luis et al. (July 2019). "Controlling Visually Guided Behavior by Holographic Recalling of Cortical Ensembles". en. In: *Cell* 178.2, 447–457.e5. ISSN: 0092-8674, 1097-4172. DOI: 10.1016/j.cell.2019.05.045 (cit. on p. 22).

Chase, Steven M, Robert E Kass, and Andrew B Schwartz (July 2012). "Behavioral and neural correlates of visuomotor adaptation observed through a brain-computer interface in primary motor cortex". In: *Journal of neurophysiology* 108.2, pp. 624–644. ISSN: 0022-3077, 1522-1598. DOI: 10.1152/jn.00371.2011 (cit. on pp. 19, 22).

Chettih, Selmaan N and Christopher D Harvey (Mar. 2019). "Single-neuron perturbations reveal feature-specific competition in V1". en. In: *Nature* 567.7748, pp. 334–340. ISSN: 0028-0836, 1476-4687. DOI: 10.1038/s41586-019-0997-6 (cit. on p. 22).

Churchland, Mark M et al. (June 2012). "Neural population dynamics during reaching". In: *Nature*. ISSN: 0028-0836. DOI: 10.1038/nature11129 (cit. on p. 2).

Duncker, L, L Driscoll, K V Shenoy, et al. (2020). "Organizing recurrent network dynamics by task-computation to enable continual learning". In: *Advances in neural information processing systems* (cit. on p. 21).

Fraser, George W and Andrew B Schwartz (2012). "Recording from the same neurons chronically in motor cortex". In: *Journal of neurophysiology* 107.7, pp. 1970–1978. ISSN: 0022-3077. DOI: 10.1152/jn.01012.2010 (cit. on p. 18).

Golub, Matthew D et al. (Mar. 2018). "Learning by neural reassociation". en. In: *Nature neuroscience*, p. 1. ISSN: 1097-6256. DOI: 10.1038/s41593-018-0095-3 (cit. on pp. 16, 19, 22).

Hatsopoulos, Nicholas G and Aaron J Suminski (Nov. 2011). "Sensing with the Motor Cortex". In: *Neuron* 72.3, pp. 477–487. ISSN: 0896-6273. DOI: 10.1016/j.neuron.2011.10.020 (cit. on p. 15).

Hennequin, Guillaume, Tim P Vogels, and Wulfram Gerstner (June 2014). "Optimal control of transient dynamics in balanced networks supports generation of complex movements". en. In: *Neuron* 82.6, pp. 1394–1406. ISSN: 0896-6273, 1097-4199. DOI: 10.1016/j.neuron.2014.04.045 (cit. on p. 21).

Herzfeld, David J et al. (Sept. 2014). "Contributions of the cerebellum and the motor cortex to acquisition and retention of motor memories". en. In: *NeuroImage* 98, pp. 147–158. ISSN: 1053-8119, 1095-9572. DOI: 10.1016/j.neuroimage.2014.04.076 (cit. on p. 22).

Howard, Ian S and David W Franklin (June 2015). "Neural Tuning Functions Underlie Both Generalization and Interference". en. In: *PloS one* 10.6, e0131268. ISSN: 1932-6203. DOI: 10.1371/journal.pone.0131268 (cit. on p. 11).

Hwang, Eun Jung, Paul M Bailey, and Richard A Andersen (Mar. 2013). "Volitional control of neural activity relies on the natural motor repertoire". en. In: *Current biology: CB* 23.5, pp. 353–361. ISSN: 0960-9822, 1879-0445. DOI: 10.1016/j.cub.2013.01.027 (cit. on p. 22).

Jarosiewicz, Beata et al. (Dec. 2008). "Functional network reorganization during learning in a brain-computer interface paradigm". In: *Proceedings of the National Academy of Sciences of the United States of America* 105.49, pp. 19486–19491. ISSN: 0027-8424, 1091-6490. DOI: 10.1073/pnas.0808113105 (cit. on pp. 19, 22).

Jun, James J et al. (Nov. 2017). "Fully integrated silicon probes for high-density recording of neural activity". en. In: *Nature* 551.7679, pp. 232–236. ISSN: 0028-0836, 1476-4687. DOI: 10.1038/nature24636 (cit. on p. 13).

Kaufman, Matthew T et al. (Mar. 2014). "Cortical activity in the null space: permitting preparation without movement". In: *Nature neuroscience* 17.3, pp. 440–448. ISSN: 1097-6256, 1546-1726. DOI: 10.1038/nn.3643 (cit. on pp. 13, 21).

Krakauer, John W et al. (Mar. 2019). "Motor Learning". en. In: *Comprehensive Physiology* 9.2, pp. 613–663. ISSN: 2040-4603. DOI: 10.1002/cphy.c170043 (cit. on pp. 18, 22).

Mante, Valerio et al. (Nov. 2013). "Context-dependent computation by recurrent dynamics in prefrontal cortex". In: *Nature* 503.7474, pp. 78–84. ISSN: 0028-0836, 1476-4687. DOI: 10.1038/nature12742 (cit. on pp. 3, 15).

Marshel, James H et al. (July 2019). "Cortical layer–specific critical dynamics triggering perception". en. In: *Science*, eaaw5202. ISSN: 0036-8075, 1095-9203. DOI: 10.1126/science.aaw5202 (cit. on p. 22).

Oby, Emily R, Matthew D Golub, et al. (July 2019). "New neural activity patterns emerge with long-term learning". en. In: *Proceedings of the National Academy of Sciences of the United States of America* 116.30, pp. 15210–15215. ISSN: 0027-8424, 1091-6490. DOI: 10.1073/pnas.1820296116 (cit. on p. 21).

Oby, Emily R and Byron M Yu (Aug. 2017). "A Path to Understanding How Motor Cortex Influences Muscle Activity". en. In: *Neuron* 95.3, pp. 476–478. ISSN: 0896-6273, 1097-4199. DOI: 10.1016/j.neuron.2017.07.018 (cit. on p. 21).

Perich, Matthew G, Juan A Gallego, and Lee E Miller (Nov. 2018). "A Neural Population Mechanism for Rapid Learning". en. In: *Neuron* 100.4, 964–976.e7. ISSN: 0896-6273, 1097-4199. DOI: 10.1016/j.neuron.2018.09.030 (cit. on p. 21).

Pesaran, Bijan, Matthew J Nelson, and Richard A Andersen (July 2006). "Dorsal premotor neurons encode the relative position of the hand, eye, and goal during reach planning". en. In: *Neuron* 51.1, pp. 125–134. ISSN: 0896-6273. DOI: 10.1016/j.neuron.2006.05.025 (cit. on p. 9).

Robinson, Nick T M et al. (Dec. 2020). "Targeted Activation of Hippocampal Place Cells Drives Memory-Guided Spatial Behavior". en. In: *Cell* 183.6, 1586–1599.e10. ISSN: 0092-8674, 1097-4172. DOI: 10.1016/j.cell.2020.09.061 (cit. on p. 22).

Rokni, Uri et al. (May 2007). "Motor learning with unstable neural representations". en. In: *Neuron* 54.4, pp. 653–666. ISSN: 0896-6273. DOI: 10.1016/j.neuron.2007.04.030 (cit. on pp. 4, 5).

Russo, Abigail A et al. (Feb. 2018). "Motor Cortex Embeds Muscle-like Commands in an Untangled Population Response". en. In: *Neuron* 97.4, 953–966.e8. ISSN: 0896-6273, 1097-4199. DOI: 10.1016/j.neuron.2018.01.004 (cit. on p. 2).

Sadtler, Patrick T et al. (Aug. 2014). "Neural constraints on learning". en. In: *Nature* 512.7515, pp. 423–426. ISSN: 0028-0836, 1476-4687. DOI: 10.1038/nature13665 (cit. on p. 21).

Sakellaridi, Sofia et al. (May 2019). "Intrinsic Variable Learning for Brain-Machine Interface Control by Human Anterior Intraparietal Cortex". en. In: *Neuron* 102.3, 694–705.e3. ISSN: 0896-6273, 1097-4199. DOI: 10.1016/j.neuron.2019.02.012 (cit. on pp. 21, 22).

Scott, S H and J F Kalaska (1997). "Reaching movements with similar hand paths but different arm orientations. I. Activity of individual cells in motor cortex". In: *Journal of neurophysiology*, pp. 826–852. ISSN: 0022-3077 (cit. on p. 9).

Smith, Maurice A, Ali Ghazizadeh, and Reza Shadmehr (June 2006). "Interacting adaptive processes with different timescales underlie short-term motor learning". en. In: *PLoS biology* 4.6, e179. ISSN: 1544-9173, 1545-7885. DOI: 10.1371/journal.pbio.0040179 (cit. on p. 11).

Stavisky, Sergey D et al. (Nov. 2018). "Brain-machine interface cursor position only weakly affects monkey and human motor cortical activity in the absence of arm movements". en. In: *Scientific reports* 8.1, p. 16357. ISSN: 2045-2322. DOI: 10.1038/s41598-018-34711-1 (cit. on p. 9).

Sussillo, David et al. (July 2015). "A neural network that finds a naturalistic solution for the production of muscle activity". In: *Nature neuroscience* 18.7, pp. 1025–1033. ISSN: 1097-6256 (cit. on p. 21).

Vyas, Saurabh, Nir Even-Chen, et al. (Mar. 2018). "Neural Population Dynamics Underlying Motor Learning Transfer". en. In: *Neuron* 97.5, 1177–1186.e3. ISSN: 0896-6273, 1097-4199. DOI: 10.1016/j.neuron.2018.01.040 (cit. on pp. 13, 19, 22).

Vyas, Saurabh, Matthew D Golub, et al. (July 2020). "Computation Through Neural Population Dynamics". en. In: *Annual review of neuroscience* 43, pp. 249–275. ISSN: 0147-006X, 1545-4126. DOI: 10.1146/annurev-neuro-092619-094115 (cit. on p. 2).

Vyas, Saurabh, Daniel J O'Shea, et al. (Feb. 2020). "Causal Role of Motor Preparation during Error-Driven Learning". en. In: *Neuron*. ISSN: 1097-4199, 0896-6273. DOI: 10.1016/j.neuron.2020.01.019 (cit. on pp. 13, 19).
