# Supplementary material for: Cortical preparatory activity indexes learned motor memories
Source: Nature. Author manuscript; Available in PMC 2023 Jan 19. (PMC9851374; doi:10.1038/s41586-021-04329-x)
Supplement: Supplementary Table 1 [file NIHMS1857847-supplement-Supplementary_Table_1.pdf]

Fig. panel	Subj.	Data counts	Error bars	P value	Statistical test	Fit
1b	U	2 example sessions				
1c	U	4 learning sessions	s.e.m. over sessions			
1c	V	3 Neuropixels sessions + 2 sets of V-probe recordings	s.e.m. over sessions			
1d	U	4 learning sessions, 3 controlsessions	s.e.m. over sessions			
1d	V	3 Neuropixels sessions + 2 sets of V-probe recordings, 3 control sessions	s.e.m. over sessions			
2a	U	2 example sessions				
2a	U	Lower inset: 4 sessions, 40 data points		8.09×10^{-14}	$F_{1,38}$ test	$R^2 = 0.77$ slope = 0.64, intercept = 0.11
2b	U	2 example sessions, 12 targets per session				
2c	U	4 learning sessions, 3 control sessions	s.e.m. over sessions			
2d	U	Plotted: 1 example session, 12 targets. Statistical test of after-learning vs. before-learning states: monkey U, 4 sessions; monkey V, 3 sessions		U: 3.22×10^{-17}; V: 1.52×10^{-13}	Wilcoxon rank-sum, two-sided	
2e	U	4 learning sessions + 3 control sessions, 12 data points (12 targets) per session	box and whisker (median, 25/75 quartiles, 5/95 quantiles)	Far vs. ctrl: 1.01×10^{-9}; Near vs. ctrl: 3.04×10^{-13}; Near vs far: 0.96	Wilcoxon rank-sum, one-sided	
2f	V	1 session, 12 targets				
2g	V	1 session, 12 targets	box and whisker (median, 25/75 quartiles, 5/95 quantiles)	Blue vs. black = 1.83×10^{-5}; Red vs. black = 1.83×10^{-5}; Blue vs. red = 0.37	Wilcoxon rank-sum, one-sided	
3a	U	1 example session				
3b	U	3 example sessions, 12 targets per session				
3c	U	3 example sessions, 12 targets per session				
3d	U	CW vs. CCW: 3 sessions, 100 resampling repeats per session	box and whisker (median, 25/75 quartiles, 5/95 quantiles)	2.34×10^{-51}	Signed-rank, one-sided	
	V	CW vs. CCW: 1 session, 300 resampling repeats total	box and whisker (median, 25/75 quartiles, 5/95 quantiles)	1.58×10^{-51}	Signed-rank, one-sided	
3d	U	Field 1 vs. field 2: 5 sessions, 100 resampling repeats per session	box and whisker (median, 25/75 quartiles, 5/95 quantiles)	1.01×10^{-116}	Signed-rank, one-sided	
4b	U	1 session, last 30 simultaneous trials vs. sequential trials		CW: 1.92×10^{-6}; CCW: 0.0038	Wilcoxon rank-sum, one-sided	
4c	U	1 session, 11 targets, 100 resamples per target	s.e.m. via bootstrap			
4d	U	1 session, 7 out of 11 targets				
4e,f	U	1 session		simultaneous: 3.73×10^{-13}; sequential: 1.51×10^{-11}	Hotelling's T2 test	
5a	U	1 example session, 12 targets				

Fig. panel	Subj.	Data counts	Error bars	P value	Statistical test	Fit
5b	U	1 example session, 12 targets				
5c	U	4 sessions, 780 trials		2.96×10^{-152}	$F_{1,779}$ test	$R^2 = 0.29$
5d	U	4 sessions, 780 trials		0.44	$F_{1,779}$ test	$R^2 = 0.0075$
5e	U	Plotted: 1 example session, 12 targets. Statistical test of washout vs. after-learning states: monkey U, 4 sessions; monkey V, 3 sessions		U: 5.92×10^{-10}, 1.36×10^{-12}, 2.87×10^{-6}, 8.33×10^{-15}; V: 5.48×10^{-13}, 3.64×10^{-10}, 3.25×10^{-8}	Hotelling's T2 test	
5f	U	4 sessions, 100 resampling repeats per session	box and whisker (median, 25/75 quartiles, 5/95 quantiles)	1.06×10^{-67}	Signed-rank, one-sided	
5f	V	3 sessions, 100 resampling repeats per session	box and whisker (median, 25/75 quartiles, 5/95 quantiles)	2.63×10^{-51}	Signed-rank, one-sided	
5g	U	4 sessions	s.e.m. over sessions	5.08×10^{-4}	Wilcoxon rank-sum, one-sided	
5h	U	4 sessions	s.e.m. over sessions	0.0021	Wilcoxon rank-sum, one-sided	
5i	U+V	4+3 sessions		learning → washout x: 0.039; learning → washout y: 0.0078; washout → re-learning x: 0.0055; washout → re-learning y: 0.0012; learning → re-learning x: 0.22; learning → re-learning y: 0.47	Signed-rank, one-sided; Signed-rank, one-sided; Wilcoxon rank-sum, one-sided; Wilcoxon rank-sum, one-sided; Signed-rank, two-sided; Signed-rank, two-sided	
5j	U+V	4+3 sessions	box and whisker (median, 25/75 quartiles, 5/95 quantiles)	U: 0.029; V: 0.05; Neural shift: 0.0078	Wilcoxon rank-sum one-sided; Signed-rank, one-sided	
5k	U	5 sessions including 2 relearning sessions, 71 neurons	Dashed lines: 0.95 confidence interval	0.017	$F_{1,5}$ test	$R^2 = 0.72$
