# Supplementary material for: Cortical preparatory activity indexes learned motor memories
Source: Nature. Author manuscript; Available in PMC 2023 Jan 19. (PMC9851374; doi:10.1038/s41586-021-04329-x)
Supplement: Supplementary Table 2 [file NIHMS1857847-supplement-Supplementary_Table_2.pdf]

**Model formulation**	**Supp. Equation**	**C.V. R^2 ± s.e.m.**	
		Monkey U	Monkey V
Change in endpoint forces	Eqn. 7	0.06 ± 0.02	0.11 ± 0.06
Changes in direction tuning	Eqn. 6	0.18 ± 0.04	0.16 ± 0.06
Changes in endpoint force tuning	Eqn. 11	0.21 ± 0.07	0.22 ± 0.05
Change in muscle forces (time-averaged)	Eqn. 12 with m_{tavg}	-0.09 ± 0.01	-0.08 ± 0.01
Change in muscle forces (PCA)	Eqn. 12 with m_{pca}	0.04 ± 0.01	0.14 ± 0.01
Postural gain changes	Eqn. 13	0.21 ± 0.07	0.18 ± 0.05
True uniform shift	Eqn. 5	0.90 ± 0.03	0.93 ± 0.02

Supplementary Table 2. Explained variance of the observed uniform shift in preparatory activity by multiple alternative models, in addition to the preferred learning-related uniform shift model. Variance explained is reported as the five-fold cross-validated R^2 computed as:

$$R^2 = 1 - \frac{\text{sum of squared error}}{\text{variance}}.$$

.
